# Functional Status After Pulmonary Rehabilitation as a Predictor of Weaning Success and Survival in Patients Requiring Prolonged Mechanical Ventilation

**DOI:** 10.3389/fmed.2021.675103

**Published:** 2021-06-02

**Authors:** Li-Ta Keng, Sheng-Kai Liang, Chi-Ping Tseng, Yueh-Feng Wen, Ping-Hsien Tsou, Chia-Hao Chang, Lih-Yu Chang, Kai-Lun Yu, Meng-Rui Lee, Jen-Chung Ko

**Affiliations:** ^1^Department of Internal Medicine, National Taiwan University Hospital Hsin-Chu Branch, Hsin-Chu, Taiwan; ^2^Department of Physical Medicine and Rehabilitation, National Taiwan University Hospital Hsin-Chu Branch, Hsin-Chu, Taiwan

**Keywords:** The de Morton Mobility Index, functional status, mortality, prolonged mechanical ventilation, rehabilitation, weaning

## Abstract

**Background:** Comprehensive rehabilitation programs are recommended for patients with prolonged mechanical ventilation (PMV) to facilitate functional recovery and ventilator weaning, but whether the functional status after rehabilitation influences outcome has not been clearly evaluated. This study aimed to investigate the association between post-rehabilitation functional status and weaning and survival outcome in PMV patients.

**Methods:** We retrospectively enrolled PMV patients admitted to the respiratory care center (RCC), a post-ICU weaning facility with protocolized rehabilitation program, from January 2016 through December 2017. Functional status was measured by the de Morton Mobility Index (DEMMI), with a cut-off value set at 20 points. The primary outcomes were the weaning status at RCC discharge and hospital survival. The secondary outcomes were overall survival and survival at 3 months after RCC discharge. We followed patients until 3 months after RCC discharge or death. Logistic and Cox regressions were performed to identify significant parameters associated with weaning success and survival.

**Results:** In total, 320 patients were enrolled. The weaning success rate was 71.6%. The survival rate at RCC discharge, hospital discharge, and 3 months after RCC discharge was 89.1, 77.5, and 66.6%, respectively. Post-rehabilitation DEMMI ≥ 20 (odds ratio [OR], 3.514; 95% confidence interval [CI], 1.436–8.598; *P* = 0.006) was the most significantly associated with weaning success. The weaning success and higher post-rehabilitation DEMMI were the two most significant independent factors associated with both hospital survival (weaning success, OR, 12.272; 95% CI, 5.281–28.517; *P* < 0.001; post-rehabilitation DEMMI ≥ 20, OR, 6.298; 95% CI, 1.302–30.477; *P* = 0.022) and survival at 3 months after RCC discharge (weaning success, OR, 38.788; 95% CI, 11.505–130.762; *P* < 0.001; post-rehabilitation DEMMI ≥ 20, OR, 4.830; 95% CI, 1.072–21.756; *P* = 0.040). Post-rehabilitation DEMMI ≥ 20 remained significantly association with overall survival at 3 months after RCC discharge (hazard ratio, 0.237; 95% CI, 0.072–0.785; *P* = 0.018).

**Conclusions:** Post-rehabilitation functional status of PMV patients was independently associated with weaning success, as well as hospital and 3-month overall survival after RCC discharge. Post-rehabilitation, but not pre-rehabilitation, functional status was a significant parameter associated with weaning success and survival in patients requiring PMV.

## Introduction

One of the leading causes for intensive care unit (ICU) admission is acute respiratory failure with mechanical ventilator use (1). Due to the advancement in critical care medicine, many patients could survive through the acute stage of critical illness. About 10–20% of ICU patients, however, may require prolonged mechanical ventilation (PMV, defined as mechanical ventilator use for more than 21 days), and they consume about 37–50% of ICU resources (2). A recent systematic review showed that age, vasopressor use, thrombocytopenia, pre-existing renal disease, acute kidney injury or dialysis-dependent renal failure, and failure to wean from ventilator were strong prognostic factors for long-term mortality in patients requiring PMV (3). Among them, failure to wean has been reported as the most important prognostic factor (4).

Pulmonary rehabilitation with early mobilization and physiotherapy has been proposed as a component of multidisciplinary strategy to promote successful weaning in patients with PMV use (5). Several studies reported benefits of pulmonary rehabilitation in PMV patients including improvement in functional status (6–8), duration of spontaneous breathing trial (6), weaning rate (7), or even mortality (7, 8). Comprehensive rehabilitation programs are therefore recommended in chronic critically ill patients to speed up functional recovery and to reduce the risk of ventilator dependence or difficult weaning (9). Studies evaluating the association between functional status after routine pulmonary rehabilitation and weaning or survival outcome in patients receiving PMV, however, remain limited. Theoretically, the functional status after rehabilitation, a parameter of response to rehabilitation, should be a more reliable and proximate predictor for the weaning or survival outcome, than the acceptance of rehabilitation itself. Only one prospective study showed the change of functional status after rehabilitation was associated with weaning and survival rates in tracheostomized patients with PMV use (10). Whether post-rehabilitation functional status is associated with weaning and survival rates in all PMV patients regardless of the status with endotracheal intubation or tracheostomy remains unknown.

The aim of this study is to evaluate the association of functional status after pulmonary rehabilitation in PMV patients and the weaning and survival outcome. We hypothesize that functional status after pulmonary rehabilitation is associated better weaning and survival rates.

## Methods

### Settings and Population

This study was conducted retrospectively in the 12-bed respiratory care center (RCC), a step-down and protocol-driven weaning facility, in the National Taiwan University Hospital Hsin-Chu branch, a university-affiliated hospital in northern Taiwan. PMV was defined as ventilator support for more than 21 days, according to the definition from the National Association for Medical Direction of Respiratory Care Consensus Conference in 2005 (11), and the regulations of the National Health Insurance in Taiwan. Patients anticipated to require PMV support were transferred to the RCC if they fulfilled the following criteria: (1) age ≥ 20 years, (2) resolved acute critical illness without multiple organ failure, (3) fraction of inspiratory oxygen ≤ 0.40 and positive end-expiratory pressure ≤ 8 cm H_2_O, and (4) stable hemodynamic conditions, with norepinephrine-equivalent dose ≤ 5 μg/min. Patients who survived but failed ventilator weaning after a 6-week stay in the RCC were transferred to long-term care facilities. The nurse-to-patient ratio in the RCC was 1:4, and one respiratory therapist is on duty during each shift. All patients transferred to the RCC received standardized respiratory therapist-driven protocol for ventilator weaning (Supplementary Figure 1). All eligible RCC patients finally requiring PMV were enrolled from January 2016 to December 2017. Only the last admission was included in the analysis if patients had been admitted to the RCC more than once during the study period. The Institutional Review Board (National Taiwan University Hospital Hsin-Chu Branch REC: 107-043-E) authority approved the study and waived the required informed consent.

### Rehabilitation Protocol and Physical Function Measurement

All patients received the same physical training program conducted by experienced physical therapists 5 sessions per week during the entire RCC stay. The standardized session of physical training program included: (1) breathing exercise (deep [with doubling tidal volume] and segmental) in semi-recumbent position (≥45°), followed by cough training, with 10 repetitions per set for 1–2 sets; (2) chest mobility exercise, containing manual technique, and arm elevation with upper trunk lateral flexion, with 10 repetitions; (3) strengthening exercise for respiratory, abdominal and upper and lower limb muscles, containing antigravity elevation and resistance band exercise (for upper limbs), or straight leg raising or bridging exercise (for lower limbs), with 10 repetitions per set for 2–3 sets; (4) range-of-motion exercises (active, passive, or active-assisted, according to individual tolerance) of upper and lower extremities, with 10 repetitions; (5) functional activity training (bed mobility such as rolling or bridging, sitting, standing, or walking with adequate ventilator and oxygenation support), with 1–2 repetitions; and (6) endurance training, containing sitting/standing for 10 min, or limbs exercise/biking for 5 min. Each session lasted for 15–50 min (as long as tolerated), with all 6 elements performed as much as possible, according to individual tolerance and program progression. In neurologically impaired patients that could not follow orders, the endurance training was not performed.

Functional status was measured by the de Morton Mobility Index (DEMMI), originally developed to assess the mobility status in hospitalized elderly patients (12). It was later found to be feasible, reliable and valid in the ICU setting (13). The raw DEMMI, ranging from 0 to 19 points, was a 15-item unidimensional measure of mobility. The ordinal scale was then Rasch converted to a 0 to 100 interval scale, with a score of 0 and 100 indicating no and full mobility, respectively. The DEMMI was measured twice in the RCC by an experience physical therapist, at RCC admission (pre-rehabilitation) and before RCC discharge (post-rehabilitation), respectively. If extubation was performed during RCC stay, the measurement of post-rehabilitation DEMMI was done just before extubation.

DEMMI cut-off level for analysis in our study was determined according to the following steps and rationale. First, the corresponding optimal cut-off values determined by Youden index point on the receiver operating characteristic (ROC) curves of post-rehabilitation DEMMI were 17.5 (area under curve [AUC], 0.547; 95% confidence interval [CI], 0.479–0.615), 11.5 (AUC, 0.638; 95% CI, 0.574–0.702), and 11.5 (AUC, 0.664; 95% CI, 0.604–0.725) for weaning success, hospital survival, and 3-month survival after RCC discharge, respectively. Also, the reported minimal clinically important difference for DEMMI ranged from 10 to 20 points (14–16). The cut-off value of DEMMI in this study was therefore set at 20 points, which was the lowest converted DEMMI score more than or equal to the above mentioned cut-off values and minimal clinically important differences for consistency and simplicity.

### Data Collection

All clinical information including survival data were obtained from patient medical records and respiratory care database. The retrieved patient information during ICU stay included baseline demographic and cormorbidities, medical or surgical type of ICU admission, cause of respiratory failure, septic shock (17), and acute respiratory distress syndrome (18) in ICU. The Acute Physiology and Chronic Health Evaluation (APACHE) II was determined at ICU admission and at the time of RCC transfer, as an assessment of the disease severity (19). The retrieved patient information in RCC included the presence of tracheostomy, body mass index, Glasgow coma scale, and the results of various laboratory tests. The weaning parameters, which included maximal inspiratory pressure (P_Imax_), maximal expiratory pressure (P_Emax_), rapid shallow breathing index (RSBI), tidal volume, and minute ventilation, were measured twice, at RCC admission (pre-rehabilitation) and before extubation or RCC discharge (post-rehabilitation), respectively. The established cut-off values for weaning evaluation were used as references, including P_Imax_: −20 cm H_2_O, P_Emax_: +30 cm H_2_O, RSBI: 105, tidal volume: 5 mL/kg, and minute ventilation, 10 L/min (20). P_Imax_ and P_Emax_ were measured by a conventional pressure gauge connected to a one-way valve for airway occlusion while the patients were placed in sitting position with temporary ventilator disconnection. For P_Imax_ measurement, the patients exhaled as far as possible and then inhaled as forcefully as possible with the pressure level maintained for at least 2 s. The patients inhaled/exhaled conversely for P_Emax_ measurement. Up to 5 consecutive efforts were performed and the highest value was recorded (21). We followed all the patients until 3 months after RCC discharge or death.

### Outcome Measurements

The primary outcome measurements were the weaning status (success or failure) at RCC discharge and survival status at hospital discharge. The secondary outcome measurements were survival at 3 months after RCC discharge and overall survival after respiratory failure. Weaning success was defined as liberation from the ventilator (both invasive and non-invasive) for more than 5 days, according to the prospective payment system of ventilator dependents' managed care by the National Health Insurance in Taiwan (22). Patients were classified as weaning failure if they failed mechanical ventilator liberation at RCC discharge, returned to the ICU for deteriorated critical illness, or died during the RCC stay.

### Statistical Analysis

Data are expressed as the mean ± standard deviation and number (%) for continuous and categorical variables, respectively. Continuous variables were compared using Student's *t*-test, and categorical variables were compared using Pearson's χ2 or Fisher's exact test, as appropriate. Multivariate logistic regression models were used to identify significant clinical factors associated with weaning success, hospital survival, and 3-month survival outcome in the study population. Kaplan–Meier curves were plotted for overall survival after respiratory failure, and the differences between patient subgroups were compared using the log-rank test. Multivariate Cox proportional-hazards model with adjustment for variables with statistical significance in the univariate analysis to identify significant clinical factors associated with overall survival after respiratory failure. Variables included in the logistic and Cox multivariate analysis were statistically significant factors in the univariate analysis. Both logistic and Cox regression analyses were performed with backward selection using the entry and stay criteria at 0.05 and 0.1, respectively. We used stepwise regression in order to manage large amount of potential predictor variables and to choose the best predictor variables from the available options. A two-sided *P*-value < 0.05 was considered statistically significant. All analyses were performed using SPSS version 18.0 for Windows (IBM Corporation, Armonk, NY, USA).

## Results

### Study Population

**Figure 1:**
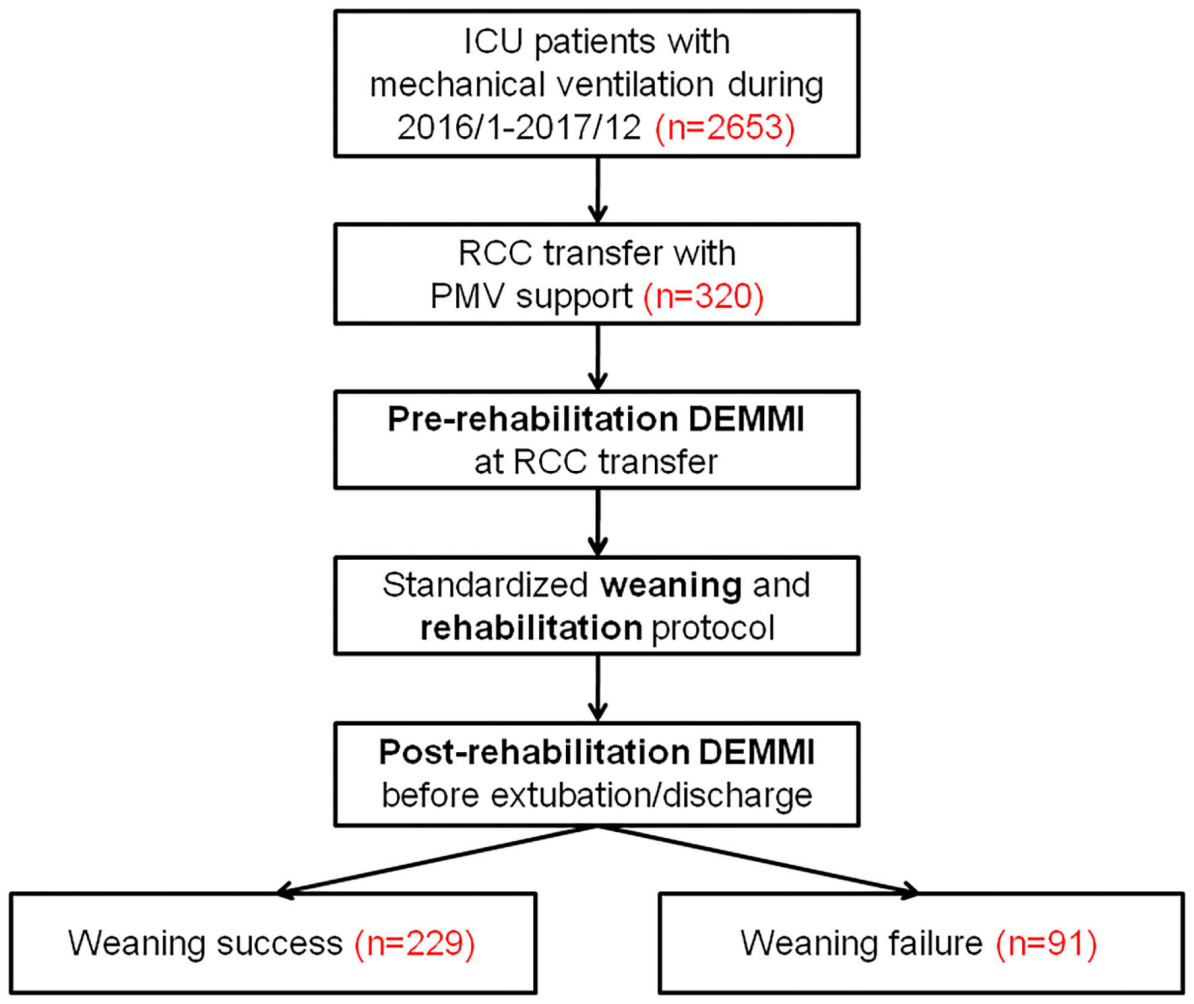
The flow diagram of the study protocol. DEMMI, the de Morton Mobility Index; ICU, intensive care unit; PMV, prolonged mechanical ventilation; RCC, respiratory care center.

Between January 2016 and December 2017, 2,653 patients were admitted to our ICUs with mechanical ventilation support. Among them, 320 patients requiring PMV support were subsequently transferred to the RCC. The mean age was 70.9 years and 202 (63.1%) were men. Pulmonary disease (40.3%) and post-operative care (25.9%) were the two most common causes of respiratory failure. The baseline characteristics and demographic features of the study population were shown in Table 1. The overall weaning success rate at RCC discharge was 71.6% (229/320). The survival rate at RCC discharge, hospital discharge, and 3 months after RCC discharge was 89.1% (285/320), 77.5% (248/320), and 66.6% (201/302), respectively. The flow diagram of the study protocol was shown in Figure 1.

**Table 1:** Clinical characteristics at baseline and intensive care unit admission.

	**Entire population**		**Weaning outcome**				***P***
			**Success**		**Failure**		
***N***	320		229		91		
**Age (years)**	70.9 ± 13.9		70.4 ± 13.9		72.1 ± 13.9		0.324
**Male sex**	202	(63.1)	142	(62.0)	60	(65.9)	0.511
**Co-morbidities**							
Coronary artery disease	69	(21.6)	51	(22.3)	18	(19.8)	0.625
Congestive heart failure	67	(20.9)	45	(19.7)	22	(24.2)	0.369
Chronic obstructive pulmonary disease	63	(19.7)	35	(15.3)	28	(30.8)	0.002
Other chronic lung disease	37	(11.6)	20	(8.7)	17	(18.7)	0.012
Diabetes mellitus	144	(45.0)	104	(45.4)	40	(44.0)	0.813
Cirrhosis	14	(4.4)	8	(3.5)	6	(6.6)	0.234
Chronic kidney disease	84	(26.3)	54	(23.6)	30	(33.0)	0.085
End-stage renal disease	28	(8.8)	18	(7.9)	10	(11.0)	0.372
Old stroke	66	(20.6)	53	(23.1)	13	(14.3)	0.077
Other neurologic disease	47	(14.7)	31	(13.5)	16	(17.6)	0.356
Cancer	48	(15.0)	30	(13.1)	18	(19.8)	0.131
**Cause of respiratory failure**							<0.001
Pulmonary	129	(40.3)	81	(35.4)	48	(52.7)	
Cardiovascular	28	(8.8)	18	(7.9)	10	(11.0)	
Neurologic	35	(10.9)	31	(13.5)	4	(4.4)	
Post-operative	83	(25.9)	71	(31.0)	12	(13.2)	
Others	45	(14.1)	28	(12.2)	17	(18.7)	
**ICU admission**							
APACHE II	20.2 ± 7.1		19.6 ± 6.7		21.9 ± 7.9		0.025
Septic shock	107	(33.4)	68	(29.7)	39	(42.9)	0.024
ARDS	15	(4.7)	9	(3.9)	6	(6.6)	0.379

*Data are presented as the mean ± standard deviation or number (%). APACHE II, Acute Physiology and Chronic Health Evaluation score; ARDS, acute respiratory distress syndrome; ICU, intensive care unit*.

### Significant Clinical Factors Associated With Weaning Success

The clinical characteristics of patients with weaning success and those with weaning failure were described in Tables 1, 2. Compared to the PMV patients with weaning failure, those with weaning success had fewer pulmonary co-rmorbidites, fewer pulmonary causes and more post-operative causes of respiratory failure, lower APACHE II score both at ICU admission and RCC transfer, a lower incidence of septic shock at ICU admission, lower creatinine level and higher platelet, hemoglobin, and albumin level at RCC transfer. While the proportion of patients with DEMMI ≥ 20 before rehabilitation did not significantly differ between these 2 groups, patients with weaning success were more likely to have post-rehabilitation DEMMI ≥ 20 than those with weaning failure (26.6 vs. 15.4%, *P* = 0.032). Similarly, while all the pre-rehabilitation weaning parameters did not differ between these 2 groups, patients with weaning success had higher post-rehabilitation P_Emax_ (66.8 vs. 46.6%, *P* = 0.002) and lower post-rehabilitation RSBI (76.8 vs. 63.0%, *P* = 0.024) than those with weaning failure. The duration of rehabilitation did not significantly differ between patients with weaning success and those with weaning failure (11.6 ± 4.9 days vs. 13.0 ± 7.8 days; *P* = 0.095), or between groups with post-rehabilitation DEMMI ≥ 20 and those with post-rehabilitation DEMMI < 20 (12.4 ± 5.6 days vs. 11.9 ± 6.0 days; *P* = 0.500). Multivariate logistic regression models showed that the absence of chronic obstructive pulmonary disease (odds ratio [OR], 0.298; 95% confidence interval [CI], 0.120–0.742; *P* = 0.009), post-operative respiratory failure (OR, 2.450; 95% CI, 1.002–5.992; *P* = 0.0496), post-rehabilitation DEMMI ≥ 20 (OR, 3.514; 95% CI, 1.436–8.598; *P* = 0.006), post-rehabilitation RSBI ≤ 105 (OR, 2.644; 95% CI, 1.290–5.419; *P* = 0.008), and higher platelet counts (increments of 10^4^/μL; OR, 1.031; 95% CI, 1.005–1.058; *P* = 0.020) were significantly associated with weaning success (Table 3).

**Table 2:** Clinical characteristics during respiratory care center hospitalization.

	**Entire population**		**Weaning outcome**				***P***
			**Success**		**Failure**		
***N***	320		229		91		
**RCC transfer**							
**APACHE II**	15.6 ± 4.8		15.1 ± 4.6		17.0 ± 5.1		0.001
**BMI (kg/m**^**2**^**)**	24.2 ± 6.0		23.9 ± 5.3		24.8 ± 7.4		0.293
**GCS**	12.2 ± 3.5		12.4 ± 3.3		11.8 ± 3.9		0.177
**Tracheostomy**	64	(20.0)	43	(18.8)	21	(23.1)	0.386
**DEMMI (pre-rehabilitation)** ≥ **20**	15	(4.7)	11	(4.8)	4	(4.4)	>0.999
**Laboratory examinations**							
Leukocytes (10^3^/μL)	10.8 ± 4.7		10.5 ± 4.4		11.3 ± 5.4		0.222
Platelets (10^3^/μL)	275 ± 150		291 ± 150		233 ± 142		0.002
Hemoglobin (g/dL)	9.7 ± 1.8		9.9 ± 1.9		9.3 ± 1.5		0.003
Albumin (g/dL)	2.8 ± 0.5		2.9 ± 0.5		2.6 ± 0.5		<0.001
Bilirubin (mg/dL)	0.9 ± 2.5		0.6 ± 0.9		1.5 ± 4.4		0.092
Creatinine (mg/dL)	1.7 ± 2.0		1.5 ± 1.8		2.1 ± 2.4		0.033
Phosphate (mg/dL)	3.7 ± 1.3		3.6 ± 1.1		3.9 ± 1.8		0.132
**Weaning parameter (pre-rehabilitation**, ***N*** **=** **264)**							
P_Imax_ ≤ −20 cm H_2_O	238	(90.2)	173	(90.6)	65	(89.0)	0.708
P_Emax_ ≥ 30 cm H_2_O	152	(57.6)	116	(60.7)	36	(49.3)	0.093
RSBI ≤ 105	169	(63.8)	122	(63.5)	47	(64.4)	0.899
Tidal volume ≥ 5 mL/kg	153	(57.7)	106	(55.2)	47	(64.4)	0.177
Minute ventilation ≤ 10 L/min	213	(80.4)	156	(81.3)	57	(78.1)	0.562
**RCC stay**							
**DEMMI (post-rehabilitation)** ≥ **20**	75	(23.4)	61	(26.6)	14	(15.4)	0.032
**Weaning parameter (post-rehabilitation**, ***N*** **=** **266)**							
P_Imax_ ≤ −20 cm H_2_O	245	(92.1)	178	(92.2)	67	(91.8)	0.904
P_Emax_ ≥ 30 cm H_2_O	163	(61.3)	129	(66.8)	34	(46.6)	0.002
RSBI ≤ 105	195	(73.0)	149	(76.8)	46	(63.0)	0.024
Tidal volume ≥ 5 mL/kg	141	(52.8)	96	(49.5)	45	(61.6)	0.076
Minute ventilation ≤ 10 L/min	217	(81.3)	158	(81.4)	59	(80.8)	0.908
**Duration of rehabilitation (days)**	12.0 ± 5.9		11.6 ± 4.9		13.0 ± 7.8		0.095

*Data are presented as mean ± standard deviation or number (%). APACHE II, Acute Physiology and Chronic Health Evaluation score; BMI, body mass index; DEMMI, the de Morton Mobility Index; GCS, Glasgow Coma Scale; P_Emax_, maximal expiratory pressure; P_Imax_, maximal inspiratory pressure; RCC, respiratory care center; RSBI, rapid shallow breath index*.

**Table 3:** Multivariate logistic regression models for significant clinical characteristics associated with weaning success at RCC discharge^*^.

**Parameters**	**β**	**SE**	**Odds ratio**	**(95% CI)**	***P***
COPD (yes vs. no)	−1.210	0.465	0.298	(0.120–0.742)	0.009
Cause of respiratory failure					
Pulmonary				1	–
Cardiovascular	−0.587	0.055	0.556	(0.187–1.650)	0.290
Neurologic	0.460	0.710	1.584	(0.394–6.363)	0.517
Post-operative	0.896	0.456	2.450	(1.002–5.992)	0.0496
Other	−0.642	0.484	0.526	(0.204–1.359)	0.185
DEMMI (post-rehabilitation, ≥ 20 vs. < 20)	1.257	0.457	3.514	(1.436–8.598)	0.006
RSBI (post-rehabilitation, ≤ 105 vs. > 105)	0.972	0.366	2.644	(1.290–5.419)	0.008
Platelets (10^4^/μL)	0.031	0.013	1.031	(1.005–1.058)	0.020
Creatinine (mg/dL)	−0.142	0.075	0.868	(0.749–1.006)	0.060

*CI, confidence interval; COPD, chronic obstructive pulmonary disease; DEMMI, the de Morton Mobility Index; RCC, respiratory care center; RSBI, rapid shallow breath index; SE, standard error*.

* *Variables with statistical significance (P < 0.05) in the univariate analyses (Tables 1, 2) were included in the multivariate logistic regression models. Backward variable selection was performed, and the criteria of P-values for entry and stay were set at 0.05 and 0.10, respectively. Details of the regression model establishment were shown in Supplementary Table 5*.

### Significant Clinical Factors Associated With Hospital Survival and 3-Month Survival After RCC Discharge

The clinical characteristics of survivors and non-survivors at hospital discharge or 3 months after RCC discharge in PMV patients are shown in Supplementary Tables 1, 2. Among 320 PMV patients, 302 (94.4%) had follow-up 3-month survival data. In detail, among 248 PMV patients who survived at hospital discharge, 201 (81.0%) survived and 29 (11.7%) died at 3 month after RCC discharge respectively, while the survival status was unavailable in the remaining 18 patients (7.3%). While the proportion of patients with DEMMI ≥ 20 before rehabilitation did not significantly differ between groups with survival and mortality at hospital discharge or 3 month after RCC discharge, the PMV survivors at hospital discharge or 3 month after RCC discharge were more likely to have post-rehabilitation DEMMI ≥ 20 than non-survivors (hospital discharge: 29.0 vs. 4.2%, *P* < 0.001; 3 months after RCC discharge: 33.3 vs. 5.9%, *P* < 0.001). Similarly, while all the pre-rehabilitation weaning parameters did not differ between survivors and non-survivors at hospital discharge or 3 months after RCC discharge, both of those survivors had higher post-rehabilitation P_Emax_ than those non-survivors. The duration of rehabilitation did not significantly differ between patients with hospital survival and those with hospital mortality (12.2 ± 5.7 days vs. 11.2 ± 6.4 days; *P* = 0.203), or between groups with survival and mortality at 3 month after RCC discharge (12.1 ± 5.9 days vs. 11.6 ± 6.2 days; *P* = 0.476). Multivariate logistic regression models showed that weaning success, post-rehabilitation DEMMI ≥ 20, and platelets were significantly associated with both hospital survival (weaning success: OR, 12.272; 95% CI, 5.281–28.517; *P* < 0.001; post-rehabilitation DEMMI: OR, 6.298; 95% CI, 1.302–30.477; *P* = 0.022; platelets: increments of 10^4^/μL; OR, 1.045; 95% CI, 1.010–1.082; *P* = 0.012) and 3-month survival after RCC discharge (weaning success: OR, 38.788; 95% CI, 11.505–130.762; *P* < 0.001; post-rehabilitation DEMMI: OR, 4.830; 95% CI, 1.072–21.756; *P* = 0.040; platelets: increments of 10^4^/μL; OR, 1.066; 95% CI, 1.018–1.117; *P* = 0.007) (Table 4).

**Table 4:** Multivariate logistic regression models for significant clinical characteristics associated with hospital survival and 3-month survival after RCC discharge^*^.

**Parameters**	**β**	**SE**	**Odds ratio (95% CI)**	***P***
**Associated with hospital survival**				
Cancer (yes vs. no)	−1.046	0.525	0.351 (0.126–0.982)	0.046
Tracheostomy (yes vs. no)	1.773	0.687	5.888 (1.533–22.615)	0.010
GCS	0.142	0.068	1.153 (1.010–1.316)	0.035
DEMMI (post-rehabilitation, ≥ 20 vs. < 20)	1.840	0.804	6.298 (1.302–30.477)	0.022
Weaning success (yes vs. no)	2.507	0.430	12.272 (5.281–28.517)	<0.001
Platelets (10^4^/μL)	0.044	0.018	1.045 (1.010–1.082)	0.012
**Associated with 3-month survival after RCC discharge**				
BMI (kg/m^2^)	0.159	0.060	1.173 (1.044–1.318)	0.007
Old stroke	−1.518	0.607	0.219 (0.067–0.721)	0.012
Cause of respiratory failure				
Pulmonary			1	–
Cardiovascular	−0.633	0.761	0.531 (0.119–2.360)	0.405
Neurologic	2.354	1.364	10.531 (0.727–152.537)	0.084
Post-operative	−0.553	0.643 0.575	(0.163–2.029)	0.390
Other	−1.279	0.817	0.278 (0.056–1.380)	0.117
Septic shock	1.098	0.569	2.999 (0.984–9.140)	0.053
GCS	0.152	0.089	1.164 (0.977–1.386)	0.089
DEMMI (post-rehabilitation, ≥ 20 vs. < 20)	1.575	0.768	4.830 (1.072–21.756)	0.040
Weaning success (yes vs. no)	3.658	0.620	38.788 (11.505–130.762)	<0.001
Platelets (10^4^/μL)	0.064	0.024	1.066 (1.018–1.117)	0.007

*BMI, body mass index; CI, confidence interval; DEMMI, the de Morton Mobility Index; GCS, Glasgow Coma Scale; RCC, respiratory care center; SE, standard error*.

* *Variables with statistical significance (P < 0.05) in the univariate analyses (Supplementary Tables 1, 2) were included in the multivariate logistic regression models. Backward variable selection was performed, and the criteria of P-values for entry and stay were set at 0.05 and 0.10, respectively. Details of the regression model establishment were shown in Supplementary Tables 6, 7*.

### Association Between Different Pre-rehabilitation and Post-rehabilitation DEMMI and Weaning and Survival Status

**Figure 2:**
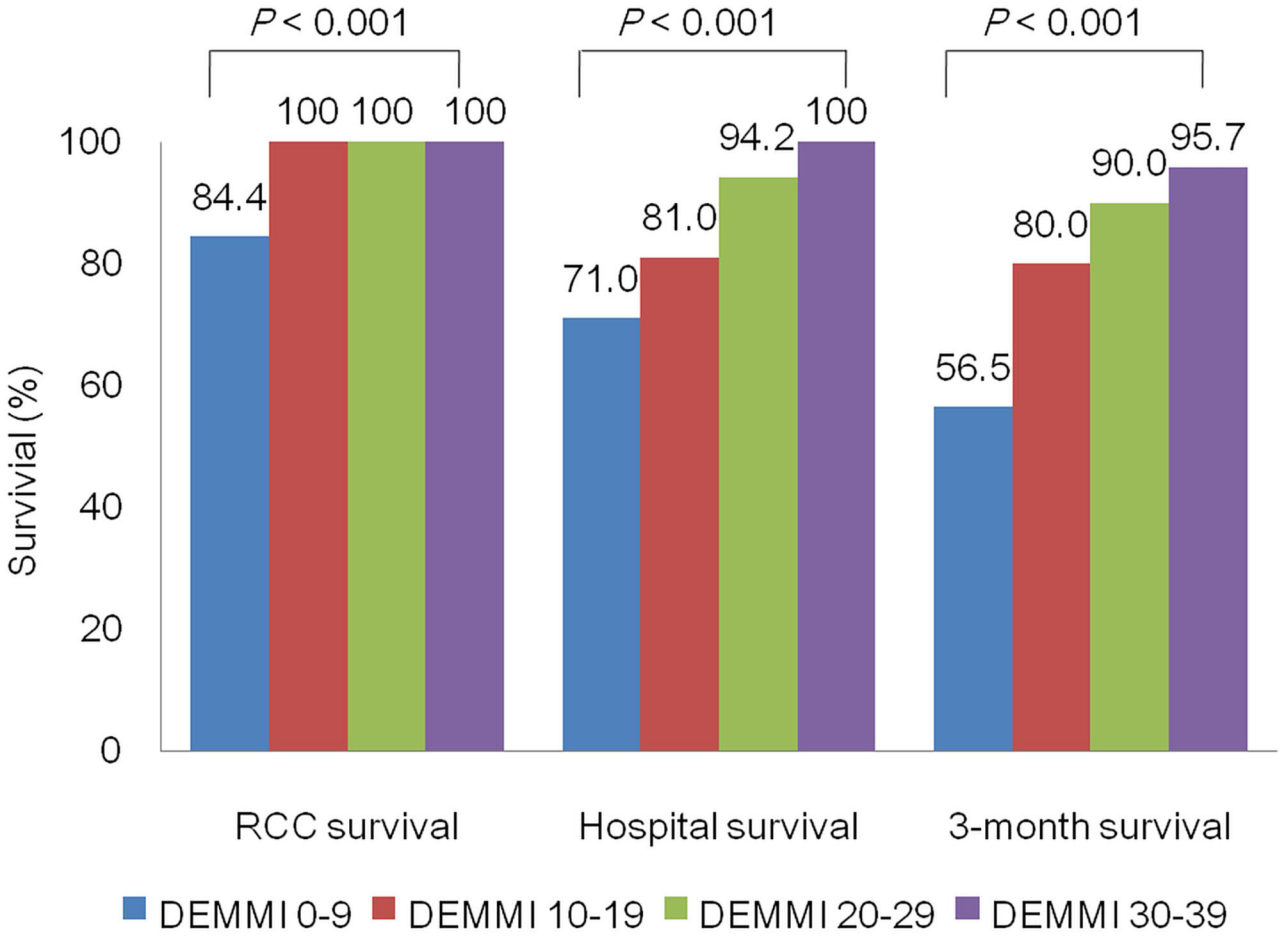
Comparison of survival status among patients of different post-rehabilitation DEMMI group. DEMMI, the de Morton Mobility Index; RCC, respiratory care center.

To further evaluate the association between various post-rehabilitation DEMMI and survival status at different time points, we divided the study population into 4 groups according to post-rehabilitation DEMMI: 0–9, 10–19, 20–29, and 30–39. The PMV patients with higher post-rehabilitation DEMMI had higher survival rate at RCC discharge, hospital discharge, and 3 months after RCC discharge (all *P* < 0.001). The 3-month survival rate after RCC discharge surpassed 90 and 95% in patients with post-rehabilitation DEMMI ≥ 20 and ≥ 30, respectively (Figure 2).

**Figure 3:**
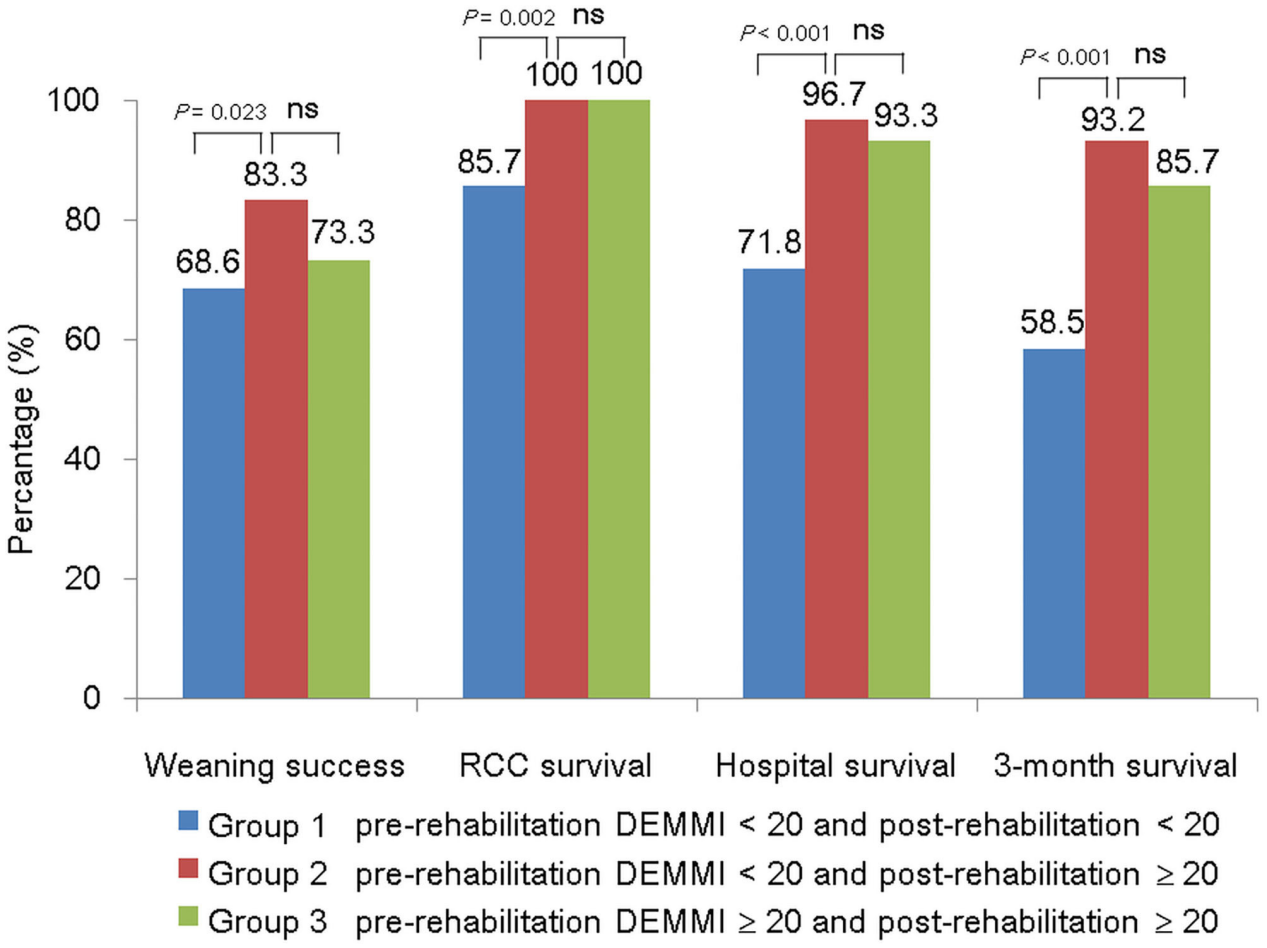
Comparison of weaning and survival status among patient groups stratified by different pre- and post-rehabilitation DEMMI combination. DEMMI, the de Morton Mobility Index; ns, not significant; RCC, respiratory care center.

We also compared different weaning and survival status among patient groups stratified by different pre- and post-rehabilitation DEMMI combination, as shown in Figure 3. All patients with pre-rehabilitation DEMMI ≥ 20 had post-rehabilitation DEMMI ≥ 20. Patients with post-rehabilitation DEMMI ≥ 20 had similar weaning and survival outcome, regardless of their corresponding pre-rehabilitation DEMMI. In the contrary, in patients with pre-rehabilitation DEMMI < 20, those who had post-rehabilitation DEMMI ≥ 20 (rehabilitation responders) had significantly higher weaning (83.3 vs. 68.6%, *P* = 0.023) and survival (100 vs. 85.7%, *P* = 0.002, at RCC discharge; 96.7 vs. 71.8%, *P* < 0.001, at hospital discharge; 93.2 vs. 58.5%, *P* < 0.001, at 3 months after RCC discharge) rate at various time points than those who still had post-rehabilitation DEMMI < 20 (rehabilitation non-responders).

Among patients with weaning failure, patients with post-rehabilitation DEMMI < 20 (respiratory care ward transfer: 30.8% [28/77]; ICU transfer: 19.8% [18/77]; death in RCC: 34.1% [31/77]) were more likely to end up with ICU transfer or death in RCC than those with post-rehabilitation DEMMI ≥ 20 (respiratory care ward transfer: 15.4% [14/14]; ICU transfer or death in RCC: 0% [0/14]) (*P* < 0.001).

### Overall Survival in Patients With Different Post-rehabilitation DEMMI

**Figure 4:**
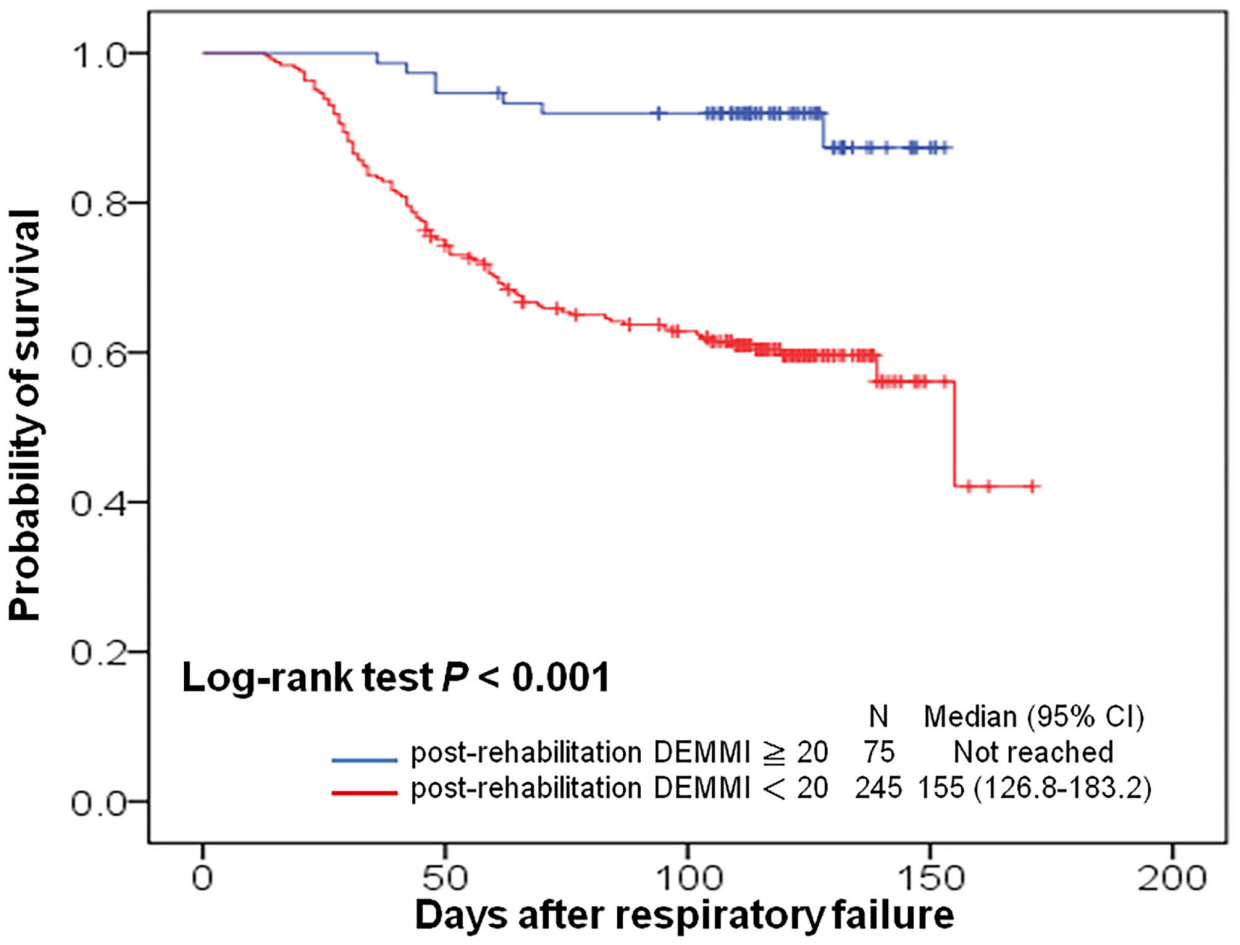
Kaplan-Meier curves for overall survival in patients with prolonged mechanical ventilation, with stratification by post-rehabilitation DEMMI. CI, confidence interval; DEMMI, the de Morton Mobility Index.

Kaplan-Meier survival curves were plotted for the different patient subgroups (Figure 4). Patients with post-rehabilitation DEMMI ≥ 20 had significantly longer overall survival than those with post-rehabilitation DEMMI < 20 (median survival: > 150 days, not reached vs. 155 days, *P* < 0.001). The duration of rehabilitation was not significantly associated with overall survival. A multivariate Cox proportional hazards model with adjusting for significant covariates showed that weaning success (hazard ratio [HR], 0.168; 95% CI, 0.096–0.293; *P* < 0.001), post-rehabilitation DEMMI ≥ 20 (HR, 0.237; 95% CI, 0.072–0.785; *P* = 0.018), and higher platelet counts (increments of 10^4^/μL; HR, 0.974; 95% CI, 0.953–0.995; *P* = 0.017) were significantly associated with better overall survival, while higher total bilirubin level (HR, 1.062; 95% CI, 1.008–1.119; *P* = 0.025), the presence of end-stage renal disease (HR, 2.368; 95% CI, 1.034–5.420; *P* = 0.041) and old stroke (HR, 1.974; 95% CI, 1.047–3.723; *P* = 0.036) were associated with worse overall survival (Table 5).

**Table 5:** Multivariate Cox regression models for significant clinical characteristics associated with overall survival after respiratory failure^*^.

**Parameters**	**Hazard ratio**	**(95% CI)**	***P***
Age (years)	1.021	(0.998–1.045)	0.080
End-stage renal disease (yes vs. no)	2.368	(1.034–5.420)	0.041
Old stroke (yes vs. no)	1.974	(1.047–3.723)	0.036
GCS	0.929	(0.856–1.008)	0.076
Platelets (10^4^/μL)	0.974	(0.953–0.995)	0.017
Bilirubin (mg/dL)	1.062	(1.008–1.119)	0.025
DEMMI (post-rehabilitation, ≥ 20 vs. < 20)	0.237	(0.072–0.785)	0.018
Weaning success (yes vs. no)	0.168	(0.096–0.293)	<0.001

*CI, confidence interval; DEMMI, the de Morton Mobility Index; GCS, Glasgow Coma Scale*.

* *Variables with statistical significance (P < 0.05) in the univariate analyses (Supplementary Table 3) were included in the multivariate Cox regression models. Backward variable selection was performed, and the criteria of P-values for entry and stay were set at 0.05 and 0.10, respectively. Details of the regression model establishment were shown in Supplementary Table 8*.

## Discussion

Our study revealed that, in patient with PMV use, post-rehabilitation DEMMI was an important prognostic factor independently associated with weaning success, hospital survival and 3-month survival after RCC discharge. Post-rehabilitation DEMMI, but not pre-rehabilitation DEMMI or the change of DEMMI after rehabilitation, could predict weaning success and short-term and mid-term survival in patients requiring PMV.

DEMMI as a functional measurement tool has been validated in inpatient population (15) and ICU population (13). Using DEMMI as functional status measurement in the post-ICU rehabilitation cohort has not yet been evaluated. In this study, post-rehabilitation functional status in PMV patients, as measured using DEMMI, showed significant correlation with weaning success, hospital survival and 3-months survival after RCC discharge. Our study showed that DEMMI could be used for functional status evaluation in post-ICU population requiring PMV and also an important prognostic factor for weaning and survival outcome.

Although previous studies evaluating the effectiveness of pulmonary rehabilitation programs on weaning rate in patients with PMV use showed conflicting results (6–8, 23), routine pulmonary rehabilitation had been adopted as a facet of multidisciplinary care for PMV patients to facilitate weaning. In this study, all enrolled PMV received standardized pulmonary rehabilitation protocol, but only those patients with higher post-rehabilitation functional status had significant higher weaning rate. Functional response after pulmonary rehabilitation, therefore, may be more important than receiving pulmonary rehabilitation itself on weaning outcome in patients requiring PMV. In patients with PMV use, underlying pulmonary derangements such as the absence of chronic obstructive pulmonary disease and the presence of post-operative cause of respiratory failure were independently associated with weaning success. But the post-rehabilitation functional status was the most important determinant independently associated with weaning success, emphasizing the significance of pulmonary rehabilitation during RCC stay.

A recent meta-analysis showed age, thrombocytopenia, vasopressor requirement, failed ventilator disconnection, preexisting kidney disease, and acute kidney injury or hemodialysis requirement as 6 important prognostic factors for long-term mortality in PMV patients (3). In another prospective observational study, weaning outcome was the most important prognostic factor for 1-year mortality in patients requiring PMV (4), which remained significant even in cancer patients with PMV use (24). In our study, the finding that weaning outcome was the most important prognostic factor for both hospital survival and 3-month survival after RCC discharge is in general agreement with previous existing literatures. Our finding that post-rehabilitation functional status is also an important prognostic factor for both hospital survival and 3-month survival after RCC discharge was rarely reported in the literature (25). Even in patients with weaning failure, all patients with post-rehabilitation DEMMI ≥ 20 were transferred alive to respiratory care wards without death or ICU transfer. Since post-rehabilitation functional status was also an independent factor for weaning success which itself had been shown to be the most important factor for mortality, the influence of post-rehabilitation functional status on survival outcome may extend beyond weaning.

In this study, several confounders and biases may exist and we have adjusted it in our final model. For instance, time of hospitalization in rehabilitation may lead to bias related to the time-dependent association. The duration of receiving rehabilitation which roughly equaled to duration of RCC stay, however, was not significantly different in patients with different outcomes regarding weaning, hospital survival, 3-month survival after RCC discharge, or overall survival. The rehabilitation duration was also similar in patients with different post-rehabilitation DEMMI status. Post-rehabilitation DEMMI ≥ 20 remained significantly association with weaning success, hospital survival, and 3-month survival after RCC discharge in the multivariate logistic regression analysis, as well as overall survival in the multivariate Cox regression analysis. The benefit of longer rehabilitation time may be offset by the underlying physiological derangement causing prolonged RCC stay. Furthermore, the frequency of DEMMI evaluation may also be a potential confounder. In our study, DEMMI was universally examined twice for all patients and this ensured that the frequency of DEMMI was similar among all patients. Understanding the functional status of PMV patients more frequently may allow physicians and physical therapists to timely adjust treatment and rehabilitation programs, and therefore improve weaning and survival outcome. The role of evaluating DEMMI may extend from a prognostic factor to guidance for individualized rehabilitation program.

A previous retrospective study showed poor functional status at admission to post-ICU acute care hospital was significantly associated with higher 1-year mortality (26). In our study, the pre-rehabilitation baseline functional status was not associated with weaning or survival outcome. In contrast, only higher post-rehabilitation functional status (rehabilitation responder) was independently associated with weaning success or survival at hospital discharge or 3 months after RCC discharge. In patients requiring PMV, the rehabilitation responders (pre-rehabilitation DEMMI < 20 and post-rehabilitation DEMMI ≥ 20) had similar weaning and survival rates as those with good functional status at RCC transfer (both pre- and post-rehabilitation DEMMI ≥ 20). This finding re-emphasized the importance of pulmonary rehabilitation and the response to this treatment in patients requiring PMV. The functional status after rehabilitation could be used as a surrogate marker for weaning and subsequent survival and it could assist both clinicians and families in understanding the prognosis during RCC stay, therefore avoiding futile aggressive treatment or introducing early palliative care. The benefit of rehabilitation may be related to treatment of muscle weakness and management of airway secretions, and therefore, improving respiratory function and decreasing bed rest complication (9). In patients with pre-rehabilitation DEMMI < 20, the rehabilitation responders (post-rehabilitation DEMMI ≥ 20) were more likely to have higher body mass index (OR, 1.108; 95% CI, 1.029–1.193; *P* = 0.006) and higher Glasgow Coma Scale (OR, 1.691; 95% CI, 1.274–2.245; *P* < 0.001) than the rehabilitation non-responders (post-rehabilitation DEMMI < 20) (Supplementary Table 4). Therefore, poor response to rehabilitation may be related to impaired nutritional or neurologic status in patients required PMV. Further researches are needed to elucidate whether response to rehabilitation could be modified by interventions targeting nutritional and neurologic components such as aggressive nutritional support and sedation/delirium protocol.

Besides weaning success and higher post-rehabilitation functional status, our study showed a higher platelet count was independently associated with both survival at hospital discharge and 3 months after RCC discharge. This finding was generally consistent with previous observational studies and meta-analysis (3, 27, 28). The underlying cause of thrombocytopenia can be multifactorial (29). The association between thrombocytopenia and mortality in patients requiring PMV may just reflect unresolved underlying disease or acute critical condition, and prophylactic platelet transfusion may increase the risk of nosocomial infection, thrombotic events, organ dysfunction, and even mortality (30).

The present study had some limitations. First, this is a single center study with retrospective nature. Second, survival status beyond 3 months after RCC discharge was not obtained. Third, the average age of the population was more than 70 years, which was higher than that in previous PMV studies (31). Our results may not be generalizable to other population.

## Conclusions

Functional status after pulmonary rehabilitation was independently associated with weaning success, hospital survival, and 3-month overall survival after RCC discharge in patients requiring PMV support. Post-rehabilitation functional status, but not pre-rehabilitation functional status, was a significant prognostic factor associated with weaning success and survival in patients requiring PMV. Physicians, therefore, should not decline or withhold rehabilitation for PMV patients who had poor performance status at initial post-ICU stage. Further studies were warranted to validate our findings.

## Data Availability Statement

The raw data supporting the conclusions of this article will be made available by the authors, without undue reservation.

## Ethics Statement

The studies involving human participants were reviewed and approved by The Institutional Review Board authority of National Taiwan University Hospital Hsin-Chu Branch (REC: 107-043-E). Written informed consent for participation was not required for this study in accordance with the national legislation and the institutional requirements.

## Author Contributions

L-TK, S-KL, C-PT, and M-RL participated in the study concept and design. C-PT, Y-FW, P-HT, C-HC, L-YC, and K-LY participated in the collection of data, statistical analysis, interpretation of data, and manuscript draft. S-KL, M-RL, and J-CK participated in manuscript revision. All authors read and approved the final manuscript.

## Conflict of Interest

S-KL received speaking honoraria from Norvatis, Pfizer, Roche, and Boehringer Ingelheim. The remaining authors declare that the research was conducted in the absence of any commercial or financial relationships that could be construed as a potential conflict of interest.

## Abbreviations

APACHE: Acute Physiology and Chronic Health Evaluation
DEMMI: the de Morton Mobility Index
ICU: intensive care unit
P_Emax_: maximal expiratory pressure
P_Imax_: maximal inspiratory pressure
PMV: prolonged mechanical ventilation
RCC: respiratory care center
RSBI: rapid shallow breathing index.

## References

[1] Bellani G Laffey JG Pham T Fan E Brochard L Esteban A. Epidemiology, patterns of care, and mortality for patients with acute respiratory distress syndrome in intensive care units in 50 countries. JAMA. (2016) 315:788–800. 10.1001/jama.2016.0291 26903337

[2] Boles JM Bion J Connors A Herridge M Marsh B Melot C. Weaning from mechanical ventilation. Eur Respir J. (2007) 29:1033–56. 10.1183/09031936.00010206 17470624

[3] Dettmer MR Damuth E Zarbiv S Mitchell JA Bartock JL Trzeciak S. Prognostic factors for long-term mortality in critically ill patients treated with prolonged mechanical ventilation: a systematic review. Crit Care Med. (2017) 45:69–74. 10.1097/CCM.0000000000002022 27618272

[4] Bigatello LM Stelfox HT Berra L Schmidt U Gettings EM. Outcome of patients undergoing prolonged mechanical ventilation after critical illness. Crit Care Med. (2007) 35:2491–7. 10.1097/01.CCM.0000287589.16724.B2 17901840

[5] Ambrosino N Vitacca M. The patient needing prolonged mechanical ventilation: a narrative review. Multidiscip Respir Med. (2018) 13:6. 10.1186/s40248-018-0118-7 29507719 PMC5831532

[6] Chiang LL Wang LY Wu CP Wu HD Wu YT. Effects of physical training on functional status in patients with prolonged mechanical ventilation. Phys Ther. (2006) 86:1271–81. 10.2522/ptj.20050036 16959675

[7] Chen YH Lin HL Hsiao HF Chou LT Kao KC Huang CC. Effects of exercise training on pulmonary mechanics and functional status in patients with prolonged mechanical ventilation. Respir Care. (2012) 57:727–34. 10.4187/respcare.01341 22152978

[8] Chen S Su CL Wu YT Wang LY Wu CP Wu HD. Physical training is beneficial to functional status and survival in patients with prolonged mechanical ventilation. J Formos Med Assoc. (2011) 110:572–9. 10.1016/j.jfma.2011.07.008 21930067

[9] Ambrosino N Venturelli E Vagheggini G Clini E. Rehabilitation, weaning and physical therapy strategies in chronic critically ill patients. Eur Respir J. (2012) 39:487–92. 10.1183/09031936.00094411 22135278

[10] Clini EM Crisafulli E Antoni FD Beneventi C Trianni L Costi S. Functional recovery following physical training in tracheotomized and chronically ventilated patients. Respir Care. (2011) 56:306–13. 10.4187/respcare.00956 21235844

[11] MacIntyre NR Epstein SK Carson S Scheinhorn D Christopher K Muldoon S. Management of patients requiring prolonged mechanical ventilation: report of a NAMDRC consensus conference. Chest. (2005) 128:3937–54. 10.1378/chest.128.6.3937 16354866

[12] de Morton NA Davidson M Keating JL. The de Morton Mobility Index (DEMMI): an essential health index for an ageing world. Health Qual Life Outcomes. (2008) 6:63. 10.1186/1477-7525-6-63 18713451 PMC2551589

[13] Sommers J Vredeveld T Lindeboom R Nollet F Engelbert RH van der Schaaf M. de Morton mobility index is feasible, reliable, and valid in patients with critical illness. Phys Ther. (2016) 96:1658–66. 10.2522/ptj.20150339 27081202

[14] de Morton NA Davidson M Keating JL. Validity, Responsiveness and the minimal clinically important difference for the De Morton Mobility Index (DEMMI) in an older acute medical population. BMC Geriatr. (2010) 10:72. 10.1186/1471-2318-10-72 20920285 PMC2958960

[15] New PW Scroggie GD Williams CM. The validity, reliability, responsiveness and minimal clinically important difference of the de Morton mobility index in rehabilitation. Disabil Rehabil. (2017) 39:1039–43. 10.1080/09638288.2016.1179800 27334796

[16] Trøstrup J Andersen H Kam CAM Magnusson SP Beyer N. Assessment of mobility in older people hospitalized for medical illness using the de Morton Mobility index and cumulated ambulation score-validity and minimal clinical important difference. J Geriatr Phys Ther. (2019) 42:153–60. 10.1519/JPT.0000000000000170 29252932 PMC6687413

[17] Rhodes A Evans LE Alhazzani W Levy MM Antonelli M Ferrer R. Surviving sepsis campaign: international guidelines for management of sepsis and septic shock: 2016. Crit Care Med. (2017) 45:486–552. 10.1097/CCM.0000000000002255 28098591

[18] ARDS Definition Task Force Ranieri VM Rubenfeld GD Thompson BT Ferguson ND Caldwell E. Acute respiratory distress syndrome: the Berlin Definition. JAMA. (2012) 307:2526–33. 10.1001/jama.2012.5669 22797452

[19] Knaus WA Draper EA Wagner DP Zimmerman JE. APACHE II: a severity of disease classification system. Crit Care Med. (1985) 13:818–29. 3928249

[20] MacIntyre N. Discontinuing mechanical ventilatory support. Chest. (2007) 132:1049–56. 10.1378/chest.06-2862 17873200

[21] Vitacca M Paneroni M Bianchi L Clini E Vianello A Ceriana P. Maximal inspiratory and expiratory pressure measurement in tracheotomised patients. Eur Respir J. (2006) 27:343–9. 10.1183/09031936.06.00002705 16452590

[22] Shih CY Hung MC Lu HM Chen L Huang SJ Wang JD. Incidence, life expectancy and prognostic factors in cancer patients under prolonged mechanical ventilation: a nationwide analysis of 5,138 cases during 1998-2007. Crit Care. (2013) 17:R144. 10.1186/cc12823 23876301 PMC4057492

[23] Yang PH Wang CS Wang YC Yang CJ Hung JY Hwang JJ. Outcome of physical therapy intervention on ventilator weaning and functional status. Kaohsiung J Med Sci. (2010) 26:366–72. 10.1016/S1607-551X(10)70060-7 20638039 PMC11915898

[24] Keng LT Chung KP Lin SY Liang SK Cheng JC Chen IC. Significant clinical factors associated with long-term mortality in critical cancer patients requiring prolonged mechanical ventilation. Sci Rep. (2017) 7:2148. 10.1038/s41598-017-02418-4 28526862 PMC5438375

[25] Hill K Dennis DM Patman SM. Relationships between mortality, morbidity, and physical function in adults who survived a period of prolonged mechanical ventilation. J Crit Care. (2013) 28:427–32. 10.1016/j.jcrc.2013.02.012 23618778

[26] Carson SS Bach PB Brzozowski L Leff A. Outcomes after long-term acute care. An analysis of 133 mechanically ventilated patients. Am J Respir Crit Care Med. (1999) 159:1568–73. 10.1164/ajrccm.159.5.9809002 10228128

[27] Carson SS Garrett J Hanson LC Lanier J Govert J Brake MC. A prognostic model for one-year mortality in patients requiring prolonged mechanical ventilation. Crit Care Med. (2008) 36:2061–9. 10.1097/CCM.0b013e31817b8925 18552692 PMC2728216

[28] Hough CL Caldwell ES Cox CE Douglas IS Kahn JM White DB. Development and validation of a mortality prediction model for patients receiving 14 days of mechanical ventilation. Crit Care Med. (2015) 43:2339–45. 10.1097/CCM.0000000000001205 26247337 PMC4788381

[29] Greinacher A Selleng S. How I evaluate and treat thrombocytopenia in the intensive care unit patient. Blood. (2016) 128:3032–42. 10.1182/blood-2016-09-693655 28034871

[30] Blumberg N Cholette JM Schmidt AE Phipps RP Spinelli SL Heal JM. Management of platelet disorders and platelet transfusions in ICU patients. Transfus Med Rev. (2017) 31:252–7. 10.1016/j.tmrv.2017.04.002 28501326

[31] Damuth E Mitchell JA Bartock JL Roberts BW Trzeciak S. Long-term survival of critically ill patientstreated with prolonged mechanical ventilation: a systematic review and meta-analysis. Lancet Respir Med. (2015) 3:544–53. 10.1016/S2213-2600(15)00150-2 26003390

