# Supplementary material for: Functional Status After Pulmonary Rehabilitation as a Predictor of Weaning Success and Survival in Patients Requiring Prolonged Mechanical Ventilation
Source: Front Med (Lausanne). 2021 Jun 2;8:675103. doi: 10.3389/fmed.2021.675103 (PMC8206270; doi:10.3389/fmed.2021.675103)
Supplement: Supplementary file 1 [file Table_1.DOC]

**Supplementary Material Table 1**. Clinical characteristics at baseline and intensive care unit admission

|  | **Status at hospital discharge** | | | | ***P*** | **Status 3 months after RCC discharge** | | | | ***P*** |
| --- | --- | --- | --- | --- | --- | --- | --- | --- | --- | --- |
|  | Survived | | Deceased | |  | Survived | | Deceased | |  |
| **N** | 248 | (77.5) | 72 | (22.5) |  | 201 | (66.6) | 101 | (33.4) |  |
| **Age (years)** | 70.1±13.8 | | 73.6±14.1 | | .066 | 69.3±13.9 | | 73.5±13.7 | | .012 |
| **Male sex** | 154 | (62.1) | 48 | (66.7) | .479 | 124 | (61.7) | 70 | (69.3) | .193 |
| **Co-morbidities** |  |  |  |  |  |  |  |  |  |  |
| Coronary artery disease | 51 | (20.6) | 18 | (25.0) | .420 | 42 | (20.9) | 22 | (21.8) | .859 |
| Congestive heart failure | 51 | (20.6) | 16 | (22.2) | .761 | 37 | (18.4) | 24 | (23.8) | .274 |
| Chronic obstructive pulmonary disease | 45 | (18.1) | 18 | (25.0) | .198 | 38 | (18.9) | 24 | (23.8) | .324 |
| Other chronic lung disease | 26 | (10.5) | 11 | (15.3) | .263 | 22 | (10.9) | 15 | (14.9) | .329 |
| Diabetes mellitus | 110 | (44.4) | 34 | (47.2) | .667 | 93 | (46.3) | 43 | (42.6) | .543 |
| Cirrhosis | 8 | (3.2) | 6 | (8.3) | .094 | 8 | (4.0) | 6 | (5.9) | .563 |
| Chronic kidney disease | 59 | (23.8) | 25 | (34.7) | .063 | 44 | (21.9) | 35 | (34.7) | .017 |
| End-stage renal disease | 18 | (7.3) | 10 | (13.9) | .080 | 12 | (6.0) | 14 | (13.9) | .021 |
| Old stroke | 48 | (19.4) | 18 | (25.0) | .297 | 34 | (16.9) | 28 | (27.7) | .028 |
| Other neurologic disease | 32 | (12.9) | 15 | (20.8) | .094 | 27 | (13.4) | 19 | (18.8) | .220 |
| Cancer | 28 | (11.3) | 20 | (27.8) | .001 | 23 | (11.4) | 22 | (21.8) | .017 |
| **Cause of respiratory failure** |  |  |  |  | .037 |  |  |  |  | .003 |
| Pulmonary | 96 | (38.7) | 33 | (45.8) |  | 75 | (37.3) | 49 | (48.5) |  |
| Cardiovascular | 19 | (7.7) | 9 | (12.5) |  | 17 | (8.5) | 11 | (10.9) |  |
| Neurologic | 32 | (12.9) | 3 | (4.2) |  | 25 | (12.4) | 6 | (5.9) |  |
| Post-operative | 70 | (28.2) | 13 | (18.1) |  | 62 | (30.8) | 15 | (14.9) |  |
| Others | 31 | (12.5) | 14 | (19.4) |  | 22 | (10.9) | 20 | (19.8) |  |
| **ICU admission** |  |  |  |  |  |  |  |  |  |  |
| APACHE II | 19.6±6.9 | | 22.6±7.5 | | .003 | 19.2±6.9 | | 22.3±7.4 | | .001 |
| Septic shock | 76 | (30.6) | 31 | (43.1) | .049 | 55 | (27.4) | 44 | (43.6) | .005 |
| ARDS | 10 | (4.0) | 5 | (6.9) | .341 | 10 | (5.0) | 5 | (5.0) | .993 |

Data are presented as the mean±standard deviation or number (%). APACHE II = Acute Physiology and Chronic Health Evaluation score; ARDS = cute respiratory distress syndrome; ICU = intensive care unit; RCC = respiratory care center.
