# Supplementary material for: Functional Status After Pulmonary Rehabilitation as a Predictor of Weaning Success and Survival in Patients Requiring Prolonged Mechanical Ventilation
Source: Front Med (Lausanne). 2021 Jun 2;8:675103. doi: 10.3389/fmed.2021.675103 (PMC8206270; doi:10.3389/fmed.2021.675103)
Supplement: Supplementary file 2 [file Table_2.DOC]

**Supplementary Material Table 2.** Clinical characteristics during respiratory care center hospitalization

|  | **Status at hospital discharge** | | | | ***P*** | **Status 3 months after RCC discharge** | | | | ***P*** |
| --- | --- | --- | --- | --- | --- | --- | --- | --- | --- | --- |
|  | Survived | | Deceased | |  | Survived | | Deceased | |  |
| **N** | 248 | (77.5) | 72 | (22.5) |  | 201 | (66.6) | 101 | (33.4) |  |
| **RCC transfer** |  |  |  | < |  |  |  |  | < |  |
| **APACHE II** | 14.9±4.6 | | 18.2±4.8 | | .001 | 14.4±4.4 | | 17.7±5.0 | | .001 |
| **BMI (kg/m^2^)** | 24.3±6.3 | | 23.5±4.2 | | .333 | 24.8±6.6 | | 23.1±4.4 | | .008 |
| **GCS** | 12.6±3.2 | | 10.9±4.3 | | .003 | 12.8±2.9 | | 11.2±4.1 | | .001 |
| **Tracheostomy** | 55 | (22.6) | 8 | (11.1) | .032 | 45 | (22.4) | 14 | (13.9) | .078 |
| **DEMMI (pre-rehabilitation)** ≥ **20** | 14 | (5.6) | 1 | (1.4) | .205 | 12 | (6.0) | 2 | (2.0) | .153 |
| **Laboratory examinations** |  |  |  |  |  |  |  |  |  |  |
| Leukocytes (10^3^/μL) | 10.48±4.25 | | 11.67±5.87  < | | .111 | 10.55±4.31 | | 11.40±5.46  < | | .140 |
| Platelets (10^3^/μL) | 294±152 | | 207±119 | | .001 | 303±154 | | 217±127 | | .001 |
| Hemoglobin (g/dL) | 9.9±1.8 | | 9.3±1.6  < | | .010 | 10.0±1.9 | | 9.3±1.6  < | | .001 |
| Albumin (g/dL) | 2.9±0.5 | | 2.6±0.5 | | .001 | 2.9±0.5 | | 2.6±0.5 | | .001 |
| Bilirubin (mg/dL) | 0.63±.090 | | 1.68±4.93 | | .084 | 0.66±.093 | | 1.35±4.20 | | .112 |
| Creatinine (mg/dL) | 1.57±1.83 | | 2.21±2.39 | | .016 | 1.43±1.70 | | 2.21±2.36 | | .004 |
| Phosphate (mg/dL) | 3.6±1.1 | | 4.0±1.8 | | .059 | 3.5±1.1 | | 4.0±1.7 | | .029 |
| **Weaning parameter (pre-rehabilitation, N=264)** |  |  |  |  |  |  |  |  |  |  |
| P_Imax_ ≤ −20 cm H2O | 177 | (88.9) | 62 | (93.8) | .250 | 144 | (90.0) | 80 | (92.0) | .614 |
| P_Emax_ ≥ 30 cm H2O | 120 | (60.3) | 32 | (49.2) | .117 | 101 | (63.1) | 45 | (51.7) | .082 |
| RSBI ≤ 105 | 123 | (61.5) | 46 | (70.8) | .117 | 102 | (63.4) | 61 | (70.1) | .284 |
| Tidal volume ≥ 5 mL/kg | 130 | (41.0) | 37 | (46.2) | .465 | 106 | (41.0) | 57 | (49.4) | .202 |
| Minute ventilation ≤ 10 L/min | 163 | (81.5) | 50 | (76.9) | .420 | 133 | (82.6) | 64 | (73.6) | .093 |
| **RCC stay** |  |  |  |  |  |  |  |  |  |  |
| **DEMMI (post-rehabilitation)** ≥ **20** | 72 | (29.0) | 3 | (4.2)  < | .001 | 67 | (33.3) | 6 | (5.9)  < | .001 |
| **Weaning parameter (post-rehabilitation, N=266)** |  |  |  |  |  |  |  |  |  |  |
| P_Imax_ ≤ −20 cm H2O | 186 | (92.1) | 59 | (92.2) | .978 | 150 | (92.0) | 80 | (93.0) | .778 |
| P_Emax_ ≥ 30 cm H2O | 132 | (65.3) | 31 | (48.4) | .016 | 116 | (71.2) | 43 | (50.0) | .001 |
| RSBI ≤ 105 | 149 | (73.4) | 46 | (71.9) | .811 | 124 | (75.6) | 61 | (70.9) | .423 |
| Tidal volume ≥ 5 mL/kg | 145 | (49.3) | 34 | (40.6) | .228 | 115 | (47.6) | 56 | (47.7) | .986 |
| Minute ventilation ≤ 10 L/min | 167 | (82.3) | 50 | (78.1)  < | .459 | 136 | (82.9) | 67 | (77.9)  < | .335 |
| **Weaning success** | 204 | (82.3) | 25 | (34.7) | .001 | 173 | (86.1) | 38 | (37.6) | .001 |
| **Duration of rehabilitation (days)** | 12.2±5.7 | | 11.2±6.4 | | .203 | 12.1±5.9 | | 11.6±6.2 | | .476 |

Data are presented as mean±standard deviation or number (%). APACHE II = Acute Physiology and Chronic Health Evaluation score; BMI = body mass index; DEMMI = the de Morton Mobility Index; GCS = Glasgow Coma Scale; P_Emax_ = maximal expiratory pressure; P_Imax_ = maximal inspiratory pressure; RCC = respiratory care center; RSBI = rapid shallow breath index.
