# Supplementary material for: Functional Status After Pulmonary Rehabilitation as a Predictor of Weaning Success and Survival in Patients Requiring Prolonged Mechanical Ventilation
Source: Front Med (Lausanne). 2021 Jun 2;8:675103. doi: 10.3389/fmed.2021.675103 (PMC8206270; doi:10.3389/fmed.2021.675103)
Supplement: Supplementary file 3 [file Table_3.DOC]

**Supplementary Material Table 3**. Univariate Cox regression models for significant clinical characteristics associated with overall survival after respiratory failure

| **Parameters** | **Hazard ratio (95% CI)** | | ***P*** |
| --- | --- | --- | --- |
| **Baseline and ICU hospitalization** |  |  |  |
| Age (years) | 1.021 | (1.005−1.037) | .009 |
| Male sex | 1.431 | (0.940−2.178) | .095 |
| Co-morbidities |  |  |  |
| Coronary artery disease | 1.108 | (0.705−1.743) | .656 |
| Congestive heart failure | 1.251 | (0.800−1.956) | .326 |
| Chronic obstructive pulmonary disease | 1.390 | (0.892−2.167) | .146 |
| Other chronic lung disease | 1.393 | (0.815−2.382) | .226 |
| Diabetes mellitus | 0.939 | (0.638−1.382) | .751 |
| Cirrhosis | 1.481 | (0.649−3.378) | .351 |
| Chronic kidney disease | 1.755 | (1.178−2.616) | .006 |
| End-stage renal disease | 1.984 | (1.141−3.499) | .015 |
| Old stroke | 1.636 | (1.064−2.514) | .025 |
| Other neurologic disease | 1.615 | (1.0002−2.607) | .0499 |
| Cancer | 1.810 | (1.129−2.902) | .014 |
| Cause of respiratory failure |  | |  |
| Pulmonary | 1 | | − |
| Cardiovascular | 0.986 | (0.513−1.895) | .966 |
| Neurologic | 0.390 | (0.137−1.109) | .069 |
| Post-operative | 0.456 | (0.160−1.011) | .056 |
| Others | 1.352 | (0.818−2.232) | .239 |
| APACHE II at ICU admission | 1.050 | (1.021−1.080) | .001 |
| Septic shock | 1.738 | (1.179−2.563) | .005 |
| ARDS | 1.253 | (0.549−2.860) | .592 |
| **RCC hospitalization** |  |  | <  <  <  < |
| APACHE II at RCC transfer | 1.105 | (1.067−1.144) | .001 |
| BMI (kg/m^2^) | 0.963 | (0.925−1.002) | .061 |
| GCS | 0.913 | (0.870−0.958) | .001 |
| Tracheostomy | 0.613 | (0.360−1.405) | .072 |
| DEMMI (pre-rehabilitation) ≥ 20 | 0.358 | (0.088−1.451) | .150 |
| Laboratory examinations |  |  |  |
| Leukocytes (10^3^/μL) | 1.032 | (0.992−1.073) | .117 |
| Platelets (10^3^/μL) | 0.965 | (0.950−0.979) | .001 |
| Hemoglobin (g/dL) | 0.835 | (0.737−0.945) | .004 |
| Albumin (g/dL) | 0.365 | (0.248−0.538) | .001 |
| Bilirubin (mg/dL) | 1.090 | (1.038−1.146) | .001 |
| Creatinine (mg/dL) | 1.147 | (1.059−1.243) | .001 |
| Phosphate (mg/dL) | 1.214 | (1.051−1.403) | .008 |
| Weaning parameter (pre-rehabilitation) |  |  |  |
| P_Imax_ ≤ −20 cm H2O | 1.035 | (0.519−2.063) | .922 |
| P_Emax_ ≥ 30 cm H2O | 0.691 | (0.456−1.045) | .080 |
| RSBI ≤ 105 | 1.351 | (0.864−2.114) | .187 |
| Tidal volume ≥ 5 mL/kg | 0.772 | (0.510−1.168) | .221 |
| Minute ventilation ≤ 10 L/min | 0.666 | (0.414−1.071) | .094  <  < |
| DEMMI (post-rehabilitation) ≥ 20 | 0.190 | (0.088−0.408) | .001 |
| Weaning parameter (post-rehabilitation) |  |  |  |
| P_Imax_ ≤ −20 cm H2O | 1.029 | (0.475−2.228) | .942 |
| P_Emax_ ≥ 30 cm H2O | 0.545 | (0.360−0.827) | .004 |
| RSBI ≤ 105 | 0.864 | (0.546−1.365) | .530 |
| Tidal volume ≥ 5 mL/kg | 1.095 | (0.721−1.662) | .671 |
| Minute ventilation ≤ 10 L/min | 0.828 | (0.498−1.377) | .467 |
| Weaning success | 0.173 | (0.116−0.256) | .001 |
| Duration of rehabilitation (days) | 0.973 | (0.940−1.008) | .125 |

APACHE II = Acute Physiology and Chronic Health Evaluation score; ARDS = acute respiratory distress syndrome; BMI = body mass index; CI = confidence interval; DEMMI = the de Morton Mobility Index; GCS = Glasgow Coma Scale; ICU = intensive care unit; P_Emax_ = maximal expiratory pressure; P_Imax_ = maximal inspiratory pressure; RCC = respiratory care center; RSBI = rapid shallow breath index.
