# Supplementary material for: Functional Status After Pulmonary Rehabilitation as a Predictor of Weaning Success and Survival in Patients Requiring Prolonged Mechanical Ventilation
Source: Front Med (Lausanne). 2021 Jun 2;8:675103. doi: 10.3389/fmed.2021.675103 (PMC8206270; doi:10.3389/fmed.2021.675103)

**Supplementary Material Table 4**. Multivariate logistic regression models for significant clinical characteristics associated with post-rehabilitation DEMMI ≥ 20 in patients who had pre-rehabilitation DEMMI < 20*

| **Parameters** | **β** | **SE** | **Odds ratio (95% CI)** | | ***P*** |
| --- | --- | --- | --- | --- | --- |
| BMI (kg/m2) | 0.103 | 0.038 | 1.108 | (1.029−1.193) | .006 |
| Other neurologic disease than old stroke | −1.913 | 1.084 | 0.148 | (0.018−1.237) | .078 |
| Cause of respiratory failure |  |  |  |  |  |
| Pulmonary |  |  | 1 | | − |
| Cardiovascular | −0.996 | 0.683 | 0.369 | (0.097−1.409) | .145 |
| Neurologic | −18.666 | 8048.599 | 0.000 | (0.000−.) | .998 |
| Post-operative | −0.030 | 0.452 | 0.970 | (0.400−2.353) | .947 |
| Other | −1.464 | 0.749 | 0.231 | (0.053−1.004) | .051 |
| APACHE II at RCC transfer | −0.103 | 0.054 | 0.902 | (0.811−1.003)  <＜ | .056 |
| GCS | 0.525 | 0.145 | 1.691 | (1.274−2.245) | .001 |
| Albumin (g/dL) | 0.776 | 0.409 | 2.173 | (0.975−4.841) | .058 |

APACHE II = Acute Physiology and Chronic Health Evaluation score; BMI = body mass index; CI = confidence interval; DEMMI = the de Morton Mobility Index; GCS = Glasgow Coma Scale; RCC = respiratory care center; SE = standard error.

*Variables with statistical significance (*P* < .05) in the univariate analyses were included in the multivariate logistic regression models. Backward variable selection was performed, and the criteria of *P* values for entry and stay were set at .05 and .10, respectively

**Supplementary Material Figure 1**. Weaning protocol.


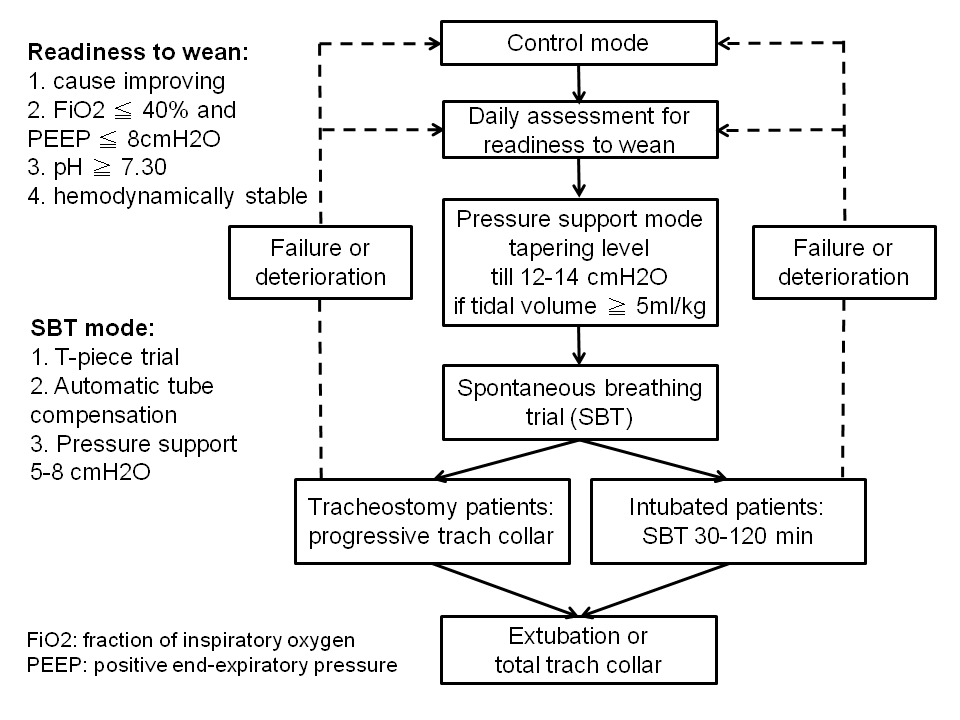

Supplement: Supplementary file 4 [file Table_4.DOC]
