# Supplementary material for: Functional Status After Pulmonary Rehabilitation as a Predictor of Weaning Success and Survival in Patients Requiring Prolonged Mechanical Ventilation
Source: Front Med (Lausanne). 2021 Jun 2;8:675103. doi: 10.3389/fmed.2021.675103 (PMC8206270; doi:10.3389/fmed.2021.675103)
Supplement: Supplementary file 5 [file Table_5.DOC]

**Supplementary Material Table 5**. Details of backward variable selection in the multivariate logistic regression models for significant clinical characteristics associated with weaning success at RCC discharge*

| **Parameters** | **β** | **SE** | **Odds ratio (95% CI)** | | ***P*** |
| --- | --- | --- | --- | --- | --- |
| **Step 1** |  |  |  |  |  |
| COPD (yes vs. no) | −1.174 | 0.475 | 0.309 | (0.122−0.785) | .014 |
| Other chronic lung disease (yes vs. no) | 0.063 | 0.561 | 1.065 | (0.355−3.196) | .911 |
| Cause of respiratory failure |  |  |  |  |  |
| Pulmonary |  |  | 1 | | − |
| Cardiovascular | −0.666 | 0.589 | 0.514 | (0.162−1.630) | .258 |
| Neurologic | 0.383 | 0.765 | 1.467 | (0.328−6.571) | .616 |
| Post-operative | 0.807 | 0.481 | 2.241 | (0.873−5.750) | .093 |
| Other | −0.680 | 0.507 | 0.507 | (0.188−1.368) | .180 |
| APACHE II at ICU admission | −0.005 | 0.026 | 0.995 | (0.945−1.047) | .845 |
| Septic shock (yes vs. no) | −0.171 | 0.378 | 0.843 | (0.402−1.766) | .650 |
| APACHE II at RCC transfer | −0.011 | 0.047 | 0.989 | (0.902−1.085) | .823 |
| Platelets (10^4^/μL) | 0.025 | 0.014 | 1.025 | (0.997−1.054) | .078 |
| Hemoglobin (g/dL) | −0.094 | 0.124 | 0.911 | (0.714−1.161) | .451 |
| Albumin (g/dL) | 0.378 | 0.393 | 1.460 | (0.676−3.155) | .336 |
| Creatinine (mg/dL) | −0.142 | 0.084 | 0.868 | (0.736−1.023) | .091 |
| DEMMI (post-rehabilitation, ≥ 20 vs. < 20) | 1.097 | 0.487 | 2.995 | (1.154−7.772) | .024 |
| P_Emax_ (post-rehabilitation, ≥ 30 vs. < 30) (cmH_2_O) | 0.414 | 0.360 | 1.513 | (0.747−3.065) | .251 |
| RSBI (post-rehabilitation, ≤ 105 vs. > 105) | 0.972 | 0.381 | 2.642 | (1.252−5.576) | .011 |
| **Step 2** |  |  |  |  |  |
| COPD (yes vs. no) | −1.166 | 0.471 | 0.312 | (0.124−0.784) | .013 |
| Cause of respiratory failure |  |  |  |  |  |
| Pulmonary |  |  | 1 | | − |
| Cardiovascular | −0.660 | 0.586 | 0.517 | (0.164−1.632) | .261 |
| Neurologic | 0.377 | 0.763 | 1.458 | (0.327−6.505) | .621 |
| Post-operative | 0.803 | 0.479 | 2.232 | (0.872−5.709) | .094 |
| Other | −0.672 | 0.502 | 0.511 | (0.191−1.365) | .180 |
| APACHE II at ICU admission | −0.005 | 0.026 | 0.995 | (0.946−1.047) | .854 |
| Septic shock (yes vs. no) | −0.173 | 0.377 | 0.841 | (0.402−1.763) | .841 |
| APACHE II at RCC transfer | −0.011 | 0.047 | 0.989 | (0.902−1.085) | .819 |
| Platelets (10^4^/μL) | 0.024 | 0.014 | 1.025 | (0.997−1.053) | .076 |
| Hemoglobin (g/dL) | −0.093 | 0.124 | 0.911 | (0.714−1.161) | .451 |
| Albumin (g/dL) | 0.387 | 0.386 | 1.472 | (0.690−3.138) | .317 |
| Creatinine (mg/dL) | −0.143 | 0.084 | 0.867 | (0.736−1.022) | .089 |
| DEMMI (post-rehabilitation, ≥ 20 vs. < 20) | 1.096 | 0.487 | 2.992 | (1.152−7.769) | .024 |
| P_Emax_ (post-rehabilitation, ≥ 30 vs. < 30) (cmH_2_O) | 0.417 | 0.359 | 1.518 | (0.751−3.068) | .245 |
| RSBI (post-rehabilitation, ≤ 105 vs. > 105) | 0.965 | 0.377 | 2.626 | (1.255−5.495) | .010 |
| **Step 3** |  |  |  |  |  |
| COPD (yes vs. no) | −1.174 | 0.469 | 0.309 | (0.123−0.775) | .012 |
| Cause of respiratory failure |  |  |  |  |  |
| Pulmonary |  |  | 1 | | − |
| Cardiovascular | −0.644 | 0.581 | 0.525 | (0.168−1.638) | .267 |
| Neurologic | 0.387 | 0.760 | 1.473 | (0.332−6.536) | .611 |
| Post-operative | 0.820 | 0.470 | 2.271 | (0.905−5.701) | .081 |
| Other | −0.675 | 0.501 | 0.509 | (0.191−1.360) | .178 |
| Septic shock (yes vs. no) | −0.172 | 0.377 | 0.842 | (0.402−1.764) | .648 |
| APACHE II at RCC transfer | −0.012 | 0.047 | 0.988 | (0.902−1.083) | .802 |
| Platelets (10^4^/μL) | 0.025 | 0.014 | 1.025 | (0.998−1.053) | .070 |
| Hemoglobin (g/dL) | −0.090 | 0.123 | 0.914 | (0.718−1.162) | .462 |
| Albumin (g/dL) | 0.392 | 0.385 | 1.480 | (0.695−3.149) | .309 |
| Creatinine (mg/dL) | −0.142 | 0.084 | 0.867 | (0.736−1.022) | .089 |
| DEMMI (post-rehabilitation, ≥ 20 vs. < 20) | 1.096 | 0.486 | 2.992 | (1.154−7.760) | .024 |
| P_Emax_ (post-rehabilitation, ≥ 30 vs. < 30) (cmH_2_O) | 0.416 | 0.359 | 1.516 | (0.750−3.065) | .246 |
| RSBI (post-rehabilitation, ≤ 105 vs. > 105) | 0.966 | 0.377 | 2.627 | (1.255−5.495) | .010 |
| **Step 4** |  |  |  |  |  |
| COPD (yes vs. no) | −1.170 | 0.469 | 0.310 | (0.124−0.778) | .013 |
| Cause of respiratory failure |  |  |  |  |  |
| Pulmonary |  |  | 1 | | − |
| Cardiovascular | −0.623 | 0.573 | 0.536 | (0.174−1.649) | .277 |
| Neurologic | 0.365 | 0.755 | 1.441 | (0.328−6.329) | .628 |
| Post-operative | 0.815 | 0.470 | 2.259 | (0.900−5.669) | .083 |
| Other | −0.665 | 0.499 | 0.514 | (0.193−1.368) | .183 |
| Septic shock (yes vs. no) | −0.178 | 0.377 | 0.837 | (0.400−1.750) | .636 |
| Platelets (10^4^/μL) | 0.025 | 0.014 | 1.025 | (0.998−1.053) | .065 |
| Hemoglobin (g/dL) | −0.085 | 0.121 | 0.919 | (0.725−1.164) | .483 |
| Albumin (g/dL) | 0.397 | 0.385 | 1.488 | (0.700−3.163) | .302 |
| Creatinine (mg/dL) | −0.150 | 0.078 | 0.860 | (0.739−1.002) | .053 |
| DEMMI (post-rehabilitation, ≥ 20 vs. < 20) | 1.118 | 0.478 | 3.058 | (1.198−7.804) | .019 |
| P_Emax_ (post-rehabilitation, ≥ 30 vs. < 30) (cmH_2_O) | 0.429 | 0.356 | 1.535 | (0.765−3.082) | .228 |
| RSBI (post-rehabilitation, ≤ 105 vs. > 105) | 0.967 | 0.376 | 2.630 | (1.258−5.500) | .010 |
| **Step 5** |  |  |  |  |  |
| COPD (yes vs. no) | −1.167 | 0.469 | 0.311 | (0.124−0.780) | .013 |
| Cause of respiratory failure |  |  |  |  |  |
| Pulmonary |  |  | 1 | | − |
| Cardiovascular | −0.608 | 0.573 | 0.545 | (0.177−1.674) | .289 |
| Neurologic | 0.393 | 0.750 | 1.481 | (0.340−6.444) | .601 |
| Post-operative | 0.833 | 0.467 | 2.299 | (0.920−5.746) | .075 |
| Other | −0.685 | 0.497 | 0.504 | (0.190−1.336) | .168 |
| Platelets (10^4^/μL) | 0.026 | 0.014 | 1.026 | (0.999−1.054) | .059 |
| Hemoglobin (g/dL) | −0.079 | 0.120 | 0.924 | (0.731−1.169) | .511 |
| Albumin (g/dL) | 0.448 | 0.369 | 1.565 | (0.758−3.228) | .226 |
| Creatinine (mg/dL) | −0.152 | 0.078 | 0.859 | (0.738−1.0004) | .051 |
| DEMMI (post-rehabilitation, ≥ 20 vs. < 20) | 1.140 | 0.476 | 3.127 | (1.231−7.946) | .017 |
| P_Emax_ (post-rehabilitation, ≥ 30 vs. < 30) (cmH_2_O) | 0.417 | 0.355 | 1.517 | (0.757−3.040) | .240 |
| RSBI (post-rehabilitation, ≤ 105 vs. > 105) | 0.969 | 0.376 | 2.634 | (1.260−5.510) | .010 |
| **Step 6** |  |  |  |  |  |
| COPD (yes vs. no) | −1.170 | 0.469 | 0.310 | (0.124−0.779) | .013 |
| Cause of respiratory failure |  |  |  |  |  |
| Pulmonary |  |  | 1 | | − |
| Cardiovascular | −0.655 | 0.567 | 0.519 | (0.171−1.578) | .248 |
| Neurologic | 0.290 | 0.731 | 1.336 | (0.319−5.600) | .692 |
| Post-operative | 0.826 | 0.466 | 2.283 | (0.916−5.692) | .076 |
| Other | −0.721 | 0.494 | 0.486 | (0.185−1.281) | .145 |
| Platelets (10^4^/μL) | 0.026 | 0.013 | 1.026 | (0.999−1.054) | .055 |
| Albumin (g/dL) | 0.390 | 0.358 | 1.477 | (0.732−2.977) | .276 |
| Creatinine (mg/dL) | −0.143 | 0.076 | 0.867 | (0.747−1.005) | .059 |
| DEMMI (post-rehabilitation, ≥ 20 vs. < 20) | 1.089 | 0.466 | 2.972 | (1.192−7.411) | .019 |
| P_Emax_ (post-rehabilitation, ≥ 30 vs. < 30) (cmH_2_O) | 0.406 | 0.354 | 1.501 | (0.751−3.002) | .251 |
| RSBI (post-rehabilitation, ≤ 105 vs. > 105) | 0.947 | 0.375 | 2.577 | (1.237−5.370) | .011 |
| **Step 7** |  |  |  |  |  |
| COPD (yes vs. no) | −1.187 | 0.470 | 0.305 | (0.121−0.767) | .012 |
| Cause of respiratory failure |  |  |  |  |  |
| Pulmonary |  |  | 1 | | − |
| Cardiovascular | −0.561 | 0.560 | 0.571 | (0.191−1.710) | .317 |
| Neurologic | 0.450 | 0.714 | 1.568 | (0.387−6.349) | .529 |
| Post-operative | 0.920 | 0.459 | 2.509 | (1.021−6.164) | .045 |
| Other | −0.687 | 0.489 | 0.503 | (0.193−1.311) | .160 |
| Platelets (10^4^/μL) | 0.028 | 0.013 | 1.029 | (1.002−1.056) | .034 |
| Creatinine (mg/dL) | −0.152 | 0.076 | 0.859 | (0.740−0.996) | .044 |
| DEMMI (post-rehabilitation, ≥ 20 vs. < 20) | 1.140 | 0.465 | 3.125 | (1.257−7.770) | .014 |
| P_Emax_ (post-rehabilitation, ≥ 30 vs. < 30) (cmH_2_O) | 0.467 | 0.348 | 1.595 | (0.807−3.155) | .180 |
| RSBI (post-rehabilitation, ≤ 105 vs. > 105) | 0.897 | 0.371 | 2.453 | (1.186−5.073) | .016 |
| **Step 8** |  |  |  |  |  |
| COPD (yes vs. no) | −1.210 | 0.465 | 0.298 | (0.120−0.742) | .009 |
| Cause of respiratory failure |  |  |  |  |  |
| Pulmonary |  |  | 1 | | − |
| Cardiovascular | −0.587 | 0.555 | 0.556 | (0.187−1.650) | .290 |
| Neurologic | 0.460 | 0.710 | 1.584 | (0.394−6.363) | .517 |
| Post-operative | 0.896 | 0.456 | 2.450 | (1.002−5.992) | .0496 |
| Other | −0.642 | 0.484 | 0.526 | (0.204−1.359) | .185 |
| Platelets (10^4^/μL) | 0.031 | 0.013 | 1.031 | (1.005−1.058) | .020 |
| Creatinine (mg/dL) | −0.142 | 0.075 | 0.868 | (0.749−1.006) | .060 |
| DEMMI (post-rehabilitation, ≥ 20 vs. < 20) | 1.257 | 0.457 | 3.514 | (1.436−8.598) | .006 |
| RSBI (post-rehabilitation, ≤ 105 vs. > 105) | 0.972 | 0.366 | 2.644 | (1.290−5.419) | .008 |

APACHE II = Acute Physiology and Chronic Health Evaluation score; CI = confidence interval; COPD = chronic obstructive pulmonary disease; DEMMI = the de Morton Mobility Index; ICU = intensive care unit; P_Emax_ = maximal expiratory pressure; RCC = respiratory care center; RSBI = rapid shallow breath index; SE = standard error.

* Variables with statistical significance (*P* < .05) in the univariate analyses (Tables 1 and 2) were included in the multivariate logistic regression models. Backward variable selection was performed, and the criteria of *P* values for entry and stay were set at .05 and .10, respectively.
