# Supplementary material for: Functional Status After Pulmonary Rehabilitation as a Predictor of Weaning Success and Survival in Patients Requiring Prolonged Mechanical Ventilation
Source: Front Med (Lausanne). 2021 Jun 2;8:675103. doi: 10.3389/fmed.2021.675103 (PMC8206270; doi:10.3389/fmed.2021.675103)
Supplement: Supplementary file 6 [file Table_6.DOC]

**Supplementary Material Table 6**. Details of backward variable selection in the multivariate logistic regression models for significant clinical characteristics associated with hospital survival*

| **Parameters** | **β** | | **SE** | **Odds ratio (95% CI)** | | ***P*** |
| --- | --- | --- | --- | --- | --- | --- |
| **Step 1** | |  |  |  |  |  |
| Cancer (yes vs. no) | | −1.142 | 0.590 | 0.319 | (0.100−1.015) | .053 |
| Cause of respiratory failure | |  |  |  |  |  |
| Pulmonary | |  |  | 1 | | − |
| Cardiovascular | | −0.466 | 0.763 | 0.627 | (0.141−2.800) | .541 |
| Neurologic | | 2.317 | 1.342 | 10.149 | (0.732−140.728) | .084 |
| Post-operative | | 0.454 | 0.616 | 1.575 | (0.471−5.273) | .461 |
| Other | | 0.428 | 0.732 | 1.535 | (0.366−6.444) | .558 |
| APACHE II at ICU admission | | −0.013 | 0.035 | 0.987 | (0.922−1.057) | .717 |
| Septic shock (yes vs. no) | | 0.496 | 0.527 | 1.643 | (0.585−4.613) | .346 |
| APACHE II at RCC transfer | | 0.038 | 0.076 | 1.039 | (0.894−1.206) | .620 |
| GCS | | 0.222 | 0.093 | 1.249 | (1.041−1.498) | .017 |
| Tracheostomy (yes vs. no) | | 2.041 | 0.771 | 7.696 | (1.699−34.865) | .008 |
| Platelets (10^4^/μL) | | 0.042 | 0.020 | 1.042 | (1.002−1.085) | .040 |
| Hemoglobin (g/dL) | | 0.110 | 0.171 | 1.116 | (0.798−1.563) | .521 |
| Albumin (g/dL) | | 0.003 | 0.474 | 1.003 | (0.396−2.538) | .995 |
| Creatinine (mg/dL) | | −0.136 | 0.104 | 0.873 | (0.712−1.071) | .193 |
| DEMMI (post-rehabilitation, ≥ 20 vs. < 20) | | 1.642 | 0.833 | 5.167 | (1.010−26.428)  < | .049 |
| Weaning success (yes vs. no) | | 2.469 | 0.480 | 11.812 | (4.613−30.242) | .001 |
| P_Emax_ (post-rehabilitation, ≥ 30 vs. < 30) (cmH_2_O) | | 0.571 | 0.471 | 1.770 | (0.704−4.453) | .225 |
| **Step 2** | |  |  |  |  |  |
| Cancer (yes vs. no) | | −1.142 | 0.590 | 0.319 | (0.100−1.015) | .053 |
| Cause of respiratory failure | |  |  |  |  |  |
| Pulmonary | |  |  | 1 | | − |
| Cardiovascular | | −0.466 | 0.758 | 0.628 | (0.142−2.773) | .539 |
| Neurologic | | 2.319 | 1.320 | 10.165 | (0.764−135.191) | .079 |
| Post-operative | | 0.455 | 0.611 | 1.576 | (0.476−5.216) | .456 |
| Other | | 0.428 | 0.732 | 1.535 | (0.366−6.441) | .558 |
| APACHE II at ICU admission | | −0.013 | 0.035 | 0.987 | (0.922−1.057) | .717 |
| Septic shock (yes vs. no) | | 0.496 | 0.508 | 1.641 | (0.607−4.442) | .329 |
| APACHE II at RCC transfer | | 0.038 | 0.076 | 1.039 | (0.895−1.205) | .619 |
| GCS | | 0.222 | 0.092 | 1.249 | (1.043−1.496) | .016 |
| Tracheostomy (yes vs. no) | | 2.042 | 0.759 | 7.703 | (1.741−34.089) | .007 |
| Platelets (10^4^/μL) | | 0.042 | 0.020 | 1.042 | (1.002−1.085) | .040 |
| Hemoglobin (g/dL) | | 0.110 | 0.171 | 1.117 | (0.799−1.560) | .518 |
| Creatinine (mg/dL) | | −0.136 | 0.104 | 0.873 | (0.712−1.071) | .193 |
| DEMMI (post-rehabilitation, ≥ 20 vs. < 20) | | 1.643 | 0.832 | 5.168 | (1.012−26.402)  < | .048 |
| Weaning success (yes vs. no) | | 2.469 | 0.477 | 11.816 | (4.636−30.111) | .001 |
| P_Emax_ (post-rehabilitation, ≥ 30 vs. < 30) (cmH_2_O) | | 0.572 | 0.467 | 1.771 | (0.708−4.427) | .221 |
| **Step 3** | |  |  |  |  |  |
| Cancer (yes vs. no) | | −1.170 | 0.586 | 0.310 | (0.098−0.978) | .046 |
| Cause of respiratory failure | |  |  |  |  |  |
| Pulmonary | |  |  | 1 | | − |
| Cardiovascular | | −0.391 | 0.731 | 0.676 | (0.161−2.833) | .593 |
| Neurologic | | 2.356 | 1.316 | 10.551 | (0.800−139.237) | .073 |
| Post-operative | | 0.520 | 0.584 | 1.682 | (0.535−5.284) | .373 |
| Other | | 0.442 | 0.726 | 1.556 | (0.375−6.460) | .542 |
| Septic shock (yes vs. no) | | 0.493 | 0.508 | 1.637 | (0.605−4.430) | .332 |
| APACHE II at RCC transfer | | 0.036 | 0.075 | 1.037 | (0.894−1.202) | .632 |
| GCS | | 0.220 | 0.092 | 1.246 | (1.041−1.492) | .016 |
| Tracheostomy (yes vs. no) | | 2.044 | 0.753 | 7.718 | (1.763−33.782) | .007 |
| Platelets (10^4^/μL) | | 0.042 | 0.020 | 1.043 | (1.003−1.085) | .035 |
| Hemoglobin (g/dL) | | 0.121 | 0.169 | 1.129 | (0.811−1.571) | .472 |
| Creatinine (mg/dL) | | −0.139 | 0.103 | 0.871 | (0.711−1.066) | .180 |
| DEMMI (post-rehabilitation, ≥ 20 vs. < 20) | | 1.645 | 0.831 | 5.182 | (1.016−26.432)  < | .048 |
| Weaning success (yes vs. no) | | 2.474 | 0.476 | 11.870 | (4.667−30.189) | .001 |
| P_Emax_ (post-rehabilitation, ≥ 30 vs. < 30) (cmH_2_O) | | 0.575 | 0.468 | 1.776 | (0.710−4.443) | .219 |
| **Step 4** | |  |  |  |  |  |
| Cancer (yes vs. no) | | −1.134 | 0.580 | 0.322 | (0.103−1.003) | .051 |
| Cause of respiratory failure | |  |  |  |  |  |
| Pulmonary | |  |  | 1 | | − |
| Cardiovascular | | −0.426 | 0.733 | 0.653 | (0.155−2.750) | .561 |
| Neurologic | | 2.294 | 1.294 | 9.911 | (0.785−125.112) | .076 |
| Post-operative | | 0.519 | 0.581 | 1.680 | (0.538−5.242) | .372 |
| Other | | 0.346 | 0.693 | 1.413 | (0.363−5.495) | .618 |
| Septic shock (yes vs. no) | | 0.547 | 0.497 | 1.728 | (0.652−4.580) | .271 |
| GCS | | 0.198 | 0.079 | 1.219 | (1.044−1.425) | .012 |
| Tracheostomy (yes vs. no) | | 2.066 | 0.750 | 7.895 | (1.815−34.345) | .006 |
| Platelets (10^4^/μL) | | 0.040 | 0.020 | 1.041 | (1.002−1.082) | .040 |
| Hemoglobin (g/dL) | | 0.110 | 0.168 | 1.116 | (0.803−1.551) | .512 |
| Creatinine (mg/dL) | | −0.117 | 0.092 | 0.890 | (0.742−1.067) | .207 |
| DEMMI (post-rehabilitation, ≥ 20 vs. < 20) | | 1.591 | 0.822 | 4.907 | (0.979−24.587)  < | .053 |
| Weaning success (yes vs. no) | | 2.468 | 0.476 | 11.801 | (4.641−30.004) | .001 |
| P_Emax_ (post-rehabilitation, ≥ 30 vs. < 30) (cmH_2_O) | | 0.569 | 0.468 | 1.767 | (0.706−4.421) | .224 |
| **Step 5** | |  |  |  |  |  |
| Cancer (yes vs. no) | | −1.179 | 0.576 | 0.307 | (0.099−0.951) | .041 |
| Cause of respiratory failure | |  |  |  |  |  |
| Pulmonary | |  |  | 1 | | − |
| Cardiovascular | | −0.350 | 0.726 | 0.705 | (0.170−2.921) | .629 |
| Neurologic | | 2.492 | 1.271 | 12.083 | (1.001−145.820) | .0499 |
| Post-operative | | 0.511 | 0.581 | 1.668 | (0.534−5.212) | .379 |
| Other | | 0.345 | 0.686 | 1.412 | (0.368−5.414) | .615 |
| Septic shock (yes vs. no) | | 0.478 | 0.486 | 1.612 | (0.622−4.179) | .325 |
| GCS | | 0.200 | 0.080 | 1.222 | (1.045−1.428) | .012 |
| Tracheostomy (yes vs. no) | | 2.065 | 0.745 | 7.888 | (1.830−33.998) | .006 |
| Platelets (10^4^/μL) | | 0.041 | 0.020 | 1.042 | (1.002−1.083) | .040 |
| Creatinine (mg/dL) | | −0.125 | 0.091 | 0.882 | (0.738−1.054) | .169 |
| DEMMI (post-rehabilitation, ≥ 20 vs. < 20) | | 1.654 | 0.820 | 5.230 | (1.048−26.103)  < | .044 |
| Weaning success (yes vs. no) | | 2.448 | 0.473 | 11.566 | (4.575−29.239) | .001 |
| P_Emax_ (post-rehabilitation, ≥ 30 vs. < 30) (cmH_2_O) | | 0.606 | 0.465 | 1.833 | (0.737−4.555) | .192 |
| **Step 6** | |  |  |  |  |  |
| Cancer (yes vs. no) | | −1.197 | 0.575 | 0.302 | (0.098−0.933) | .038 |
| Cause of respiratory failure | |  |  |  |  |  |
| Pulmonary | |  |  | 1 | | − |
| Cardiovascular | | −0.459 | 0.716 | 0.632 | (0.155−2.572) | .522 |
| Neurologic | | 2.280 | 1.232 | 9.774 | (0.874−109.366) | .064 |
| Post-operative | | 0.438 | 0.574 | 1.550 | (0.503−4.778) | .446 |
| Other | | 0.354 | 0.682 | 1.423 | (0.374−5.422) | .604 |
| GCS | | 0.202 | 0.079 | 1.224 | (1.048−1.430) | .011 |
| Tracheostomy (yes vs. no) | | 2.035 | 0.738 | 7.655 | (1.803−32.506) | .006 |
| Platelets (10^4^/μL) | | 0.037 | 0.019 | 1.037 | (0.9995−1.076) | .053 |
| Creatinine (mg/dL) | | −0.114 | 0.090 | 0.892 | (0.748−1.065) | .208 |
| DEMMI (post-rehabilitation, ≥ 20 vs. < 20) | | 1.594 | 0.824 | 4.922 | (0.978−24.770)  < | .053 |
| Weaning success (yes vs. no) | | 2.373 | 0.459 | 10.730 | (4.363−26.388) | .001 |
| P_Emax_ (post-rehabilitation, ≥ 30 vs. < 30) (cmH_2_O) | | 0.636 | 0.463 | 1.889 | (0.763−4.680) | .169 |
| **Step 7** | |  |  |  |  |  |
| Cancer (yes vs. no) | | −1.062 | 0.560 | 0.346 | (0.115−1.037) | .058 |
| Cause of respiratory failure | |  |  |  |  |  |
| Pulmonary | |  |  | 1 | | − |
| Cardiovascular | | −0.494 | 0.716 | 0.610 | (0.150−2.484) | .490 |
| Neurologic | | 2.344 | 1.244 | 10.423 | (0.909−119.474) | .060 |
| Post-operative | | 0.318 | 0.562 | 1.375 | (0.457−4.134) | .571 |
| Other | | 0.262 | 0.671 | 1.300 | (0.349−4.839) | .696 |
| GCS | | 0.190 | 0.078 | 1.209 | (1.037−1.409) | .015 |
| Tracheostomy (yes vs. no) | | 2.073 | 0.728 | 7.952 | (1.908−33.140) | .004 |
| Platelets (10^4^/μL) | | 0.038 | 0.019 | 1.038 | (1.001−1.077) | .043 |
| DEMMI (post-rehabilitation, ≥ 20 vs. < 20) | | 1.655 | 0.817 | 5.231 | (1.055−25.931)  < | .043 |
| Weaning success (yes vs. no) | | 2.450 | 0.459 | 11.584 | (4.715−28.464) | .001 |
| P_Emax_ (post-rehabilitation, ≥ 30 vs. < 30) (cmH_2_O) | | 0.526 | 0.453 | 1.692 | (0.696−4.112) | .246 |
| **Step 8** |  | |  |  |  |  |
| Cancer (yes vs. no) | −1.064 | | 0.548 | 0.345 | (0.118−1.010) | .052 |
| Cause of respiratory failure |  | |  |  |  |  |
| Pulmonary |  | |  | 1 | | − |
| Cardiovascular | −0.567 | | 0.712 | 0.567 | (0.140−2.291) | .426 |
| Neurologic | 2.536 | | 1.266 | 12.630 | (1.057−150.885) | .045 |
| Post-operative | 0.257 | | 0.552 | 1.293 | (0.438−3.818) | .642 |
| Other | 0.326 | | 0.654 | 1.386 | (0.385−4.990) | .617 |
| GCS | 0.206 | | 0.076 | 1.229 | (1.058−1.428) | .007 |
| Tracheostomy (yes vs. no) | 1.965 | | 0.713 | 7.132 | (1.763−28.848) | .006 |
| Platelets (10^4^/μL) | 0.039 | | 0.018 | 1.039 | (1.003−1.077) | .034 |
| DEMMI (post-rehabilitation, ≥ 20 vs. < 20) | 1.771 | | 0.813 | 5.878 | (1.195−28.914)  < | .029 |
| Weaning success (yes vs. no) | 2.492 | | 0.457 | 12.080 | (4.937−29.560) | .001 |
| **Step 9** |  | |  |  |  |  |
| Cancer (yes vs. no) | −1.046 | | 0.525 | 0.351 | (0.126−0.982) | .046 |
| GCS | 0.142 | | 0.068 | 1.153 | (1.010−1.316) | .035 |
| Tracheostomy (yes vs. no) | 1.773 | | 0.687 | 5.888 | (1.533−22.615) | .010 |
| Platelets (10^4^/μL) | 0.044 | | 0.018 | 1.045 | (1.010−1.082) | .012 |
| DEMMI (post-rehabilitation, ≥ 20 vs. < 20) | 1.840 | | 0.804 | 6.298 | (1.302−30.477)  < | .022 |
| Weaning success (yes vs. no) | 2.507 | | 0.430 | 12.272 | (5.281−28.517) | .001 |

APACHE II = Acute Physiology and Chronic Health Evaluation score; CI = confidence interval; DEMMI = the de Morton Mobility Index; GCS = Glasgow Coma Scale; ICU = intensive care unit; P_Emax_ = maximal expiratory pressure; RCC = respiratory care center; SE = standard error.

* Variables with statistical significance (*P* < .05) in the univariate analyses (Supplementary Material Tables 1 and 2) were included in the multivariate logistic regression models. Backward variable selection was performed, and the criteria of *P* values for entry and stay were set at .05 and .10, respectively.
