# Supplementary material for: Functional Status After Pulmonary Rehabilitation as a Predictor of Weaning Success and Survival in Patients Requiring Prolonged Mechanical Ventilation
Source: Front Med (Lausanne). 2021 Jun 2;8:675103. doi: 10.3389/fmed.2021.675103 (PMC8206270; doi:10.3389/fmed.2021.675103)
Supplement: Supplementary file 7 [file Table_7.DOC]

**Supplementary Material Table 7**. Details of backward variable selection in the multivariate logistic regression models for significant clinical characteristics associated with 3-month survival after RCC discharge*

| **Parameters** | **β** | | **SE** | **Odds ratio (95% CI)** | | ***P*** |
| --- | --- | --- | --- | --- | --- | --- |
| **Step 1** | |  |  |  |  |  |
| Age (years) | | −0.035 | 0.027 | 0.966 | (0.915−1.019) | .201 |
| Chronic kidney disease (yes vs. no) | | −0.528 | 0.822 | 0.590 | (0.118−2.952) | .520 |
| End-stage renal disease (yes vs. no) | | 1.419 | 1.449 | 4.134 | (0.241−70.774) | .327 |
| Old stroke (yes vs. no) | | −1.175 | 0.701 | 0.309 | (0.078−1.220) | .094 |
| Cancer (yes vs. no) | | −0.067 | 0.771 | 0.935 | (0.206−4.237) | .931 |
| Cause of respiratory failure | |  |  |  |  |  |
| Pulmonary | |  |  | 1 | | − |
| Cardiovascular | | −0.640 | 0.918 | 0.527 | (0.087−3.188) | .486 |
| Neurologic | | 1.508 | 1.351 | 4.519 | (0.320−63.806) | .264 |
| Post-operative | | −0.752 | 0.750 | 0.471 | (0.108−2.052) | .316 |
| Other | | −1.799 | 1.022 | 0.165 | (0.022−1.227) | .078 |
| APACHE II at ICU admission | | −0.018 | 0.045 | 0.983 | (0.900−1.073) | .695 |
| Septic shock (yes vs. no) | | 1.473 | 0.710 | 4.362 | (1.084−17.553) | .038 |
| APACHE II at RCC transfer | | 0.156 | 0.100 | 1.169 | (0.962−1.420) | .117 |
| BMI (kg/m2) | | 0.205 | 0.077 | 1.227 | (1.056−1.427) | .008 |
| GCS | | 0.257 | 0.115 | 1.293 | (1.031−1.621) | .026 |
| Platelets (10^4^/μL) | | 0.063 | 0.026 | 1.065 | (1.012−1.121) | .016 |
| Hemoglobin (g/dL) | | 0.227 | 0.202 | 1.225 | (0.845−1.863) | .261 |
| Albumin (g/dL) | | 0.551 | 0.559 | 1.736 | (0.580−5.193) | .324 |
| Creatinine (mg/dL) | | −0.405 | 0.267 | 0.667 | (0.395−1.126) | .129 |
| Phosphate (mg/dL) | | 0.041 | 0.297 | 1.042 | (0.582−1.867) | .889 |
| DEMMI (post-rehabilitation, ≥ 20 vs. < 20) | | 1.412 | 0.807 | 4.105 | (0.844−19.972) | .080 |
| P_Emax_ (post-rehabilitation, ≥ 30 vs. < 30) (cmH_2_O) | | 0.717 | 0.546 | 2.049 | (0.702−5.976)  < | .189 |
| Weaning success (yes vs. no) | | 3.903 | 0.755 | 49.552 | (11.290−217.484) | .001 |
| **Step 2** | |  |  |  |  |  |
| Age (years) | | −0.035 | 0.027 | 0.966 | (0.915−1.019) | .200 |
| Chronic kidney disease (yes vs. no) | | −0.522 | 0.818 | 0.594 | (0.119−2.951) | .524 |
| End-stage renal disease (yes vs. no) | | 1.423 | 1.453 | 4.151 | (0.240−71.679) | .327 |
| Old stroke (yes vs. no) | | −1.172 | 0.700 | 0.310 | (0.079−1.220) | .094 |
| Cause of respiratory failure | |  |  |  |  |  |
| Pulmonary | |  |  | 1 | | − |
| Cardiovascular | | −0.631 | 0.912 | 0.532 | (0.089−3.181) | .489 |
| Neurologic | | 1.524 | 1.342 | 4.589 | (0.330−63.729) | .256 |
| Post-operative | | −0.749 | 0.750 | 0.473 | (0.109−2.056) | .318 |
| Other | | −1.804 | 1.018 | 0.165 | (0.022−1.211) | .076 |
| APACHE II at ICU admission | | −0.018 | 0.044 | 0.982 | (0.900−1.071) | .681 |
| Septic shock (yes vs. no) | | 1.485 | 0.696 | 4.417 | (1.128−17.290) | .033 |
| APACHE II at RCC transfer | | 0.156 | 0.099 | 1.168 | (0.961−1.420) | .118 |
| BMI (kg/m2) | | 0.206 | 0.076 | 1.228 | (1.057−1.426) | .007 |
| GCS | | 0.258 | 0.115 | 1.294 | (1.033−1.621) | .025 |
| Platelets (10^4^/μL) | | 0.063 | 0.026 | 1.065 | (1.012−1.121) | .015 |
| Hemoglobin (g/dL) | | 0.228 | 0.201 | 1.256 | (0.846−1.863) | .258 |
| Albumin (g/dL) | | 0.552 | 0.559 | 1.736 | (0.580−5.195) | .324 |
| Creatinine (mg/dL) | | −0.405 | 0.268 | 0.667 | (0.395−1.127) | .130 |
| Phosphate (mg/dL) | | 0.042 | 0.298 | 1.043 | (0.582−1.869) | .888 |
| DEMMI (post-rehabilitation, ≥ 20 vs. < 20) | | 1.408 | 0.805 | 4.088 | (0.843−19.809) | .080 |
| P_Emax_ (post-rehabilitation, ≥ 30 vs. < 30) (cmH_2_O) | | 0.711 | 0.541 | 2.036 | (0.705−5.882)  < | .189 |
| Weaning success (yes vs. no) | | 3.918 | 0.737 | 50.285 | (11.862−213.166) | .001 |
| **Step 3** | |  |  |  |  |  |
| Age (years) | | −0.035 | 0.027 | 0.966 | (0.915−1.019) | .200 |
| Chronic kidney disease (yes vs. no) | | −0.525 | 0.818 | 0.591 | (0.119−2.937) | .521 |
| End-stage renal disease (yes vs. no) | | 1.346 | 1.345 | 3.844 | (0.275−53.685) | .317 |
| Old stroke (yes vs. no) | | −1.185 | 0.694 | 0.306 | (0.078−1.193) | .088 |
| Cause of respiratory failure | |  |  |  |  |  |
| Pulmonary | |  |  | 1 | | − |
| Cardiovascular | | −0.617 | 0.908 | 0.540 | (0.091−3.196) | .497 |
| Neurologic | | 1.547 | 1.332 | 4.699 | (0.345−63.994) | .246 |
| Post-operative | | −0.754 | 0.750 | 0.471 | (0.108−2.046) | .315 |
| Other | | −1.822 | 1.013 | 0.162 | (0.022−1.178) | .072 |
| APACHE II at ICU admission | | −0.018 | 0.044 | 0.982 | (0.901−1.071) | .689 |
| Septic shock (yes vs. no) | | 1.495 | 0.693 | 4.458 | (1.145−17.352) | .031 |
| APACHE II at RCC transfer | | 0.154 | 0.098 | 1.166 | (0.962−1.414) | .118 |
| BMI (kg/m2) | | 0.206 | 0.076 | 1.229 | (1.058−1.427) | .007 |
| GCS | | 0.256 | 0.114 | 1.292 | (1.032−1.617) | .025 |
| Platelets (10^4^/μL) | | 0.064 | 0.026 | 1.066 | (1.014−1.121) | .013 |
| Hemoglobin (g/dL) | | 0.224 | 0.200 | 1.251 | (0.846−1.851) | .261 |
| Albumin (g/dL) | | 0.570 | 0.544 | 1.769 | (0.609−5.132) | .294 |
| Creatinine (mg/dL) | | −0.381 | 0.202 | 0.683 | (0.460−1.015) | .060 |
| DEMMI (post-rehabilitation, ≥ 20 vs. < 20) | | 1.402 | 0.803 | 4.063 | (0.842−19.603) | .081 |
| P_Emax_ (post-rehabilitation, ≥ 30 vs. < 30) (cmH_2_O) | | 0.709 | 0.541 | 2.032 | (0.703−5.872)  < | .190 |
| Weaning success (yes vs. no) | | 3.917 | 0.738 | 50.260 | (11.825−213.623) | .001 |
| **Step 4** | |  |  |  |  |  |
| Age (years) | | −0.036 | 0.027 | 0.964 | (0.915−1.017) | .177 |
| Chronic kidney disease (yes vs. no) | | −0.591 | 0.803 | 0.554 | (0.115−2.675) | .462 |
| End-stage renal disease (yes vs. no) | | 1.349 | 1.341 | 3.852 | (0.278−53.364) | .315 |
| Old stroke (yes vs. no) | | −1.206 | 0.690 | 0.299 | (0.077−1.158) | .081 |
| Cause of respiratory failure | |  |  |  |  |  |
| Pulmonary | |  |  | 1 | | − |
| Cardiovascular | | −0.518 | 0.877 | 0.595 | (0.107−3.322) | .554 |
| Neurologic | | 1.544 | 1.324 | 4.684 | (0.350−62.703) | .243 |
| Post-operative | | −0.739 | 0.753 | 0.477 | (0.109−2.090) | .326 |
| Other | | −1.839 | 1.006 | 0.159 | (0.022−1.141) | .067 |
| Septic shock (yes vs. no) | | 1.497 | 0.693 | 4.468 | (1.148−17.389) | .031 |
| APACHE II at RCC transfer | | 0.154 | 0.098 | 1.167 | (0.962−1.415) | .117 |
| BMI (kg/m2) | | 0.207 | 0.077 | 1.230 | (1.058−1.430) | .007 |
| GCS | | 0.253 | 0.114 | 1.288 | (1.031−1.609) | .026 |
| Platelets (10^4^/μL) | | 0.065 | 0.025 | 1.067 | (1.015−1.122) | .010 |
| Hemoglobin (g/dL) | | 0.235 | 0.198 | 1.266 | (0.858−1.867) | .235 |
| Albumin (g/dL) | | 0.602 | 0.541 | 1.825 | (0.632−5.266) | .266 |
| Creatinine (mg/dL) | | −0.378 | 0.201 | 0.685 | (0.462−1.017) | .060 |
| DEMMI (post-rehabilitation, ≥ 20 vs. < 20) | | 1.415 | 0.808 | 4.116 | (0.846−20.039) | .080 |
| P_Emax_ (post-rehabilitation, ≥ 30 vs. < 30) (cmH_2_O) | | 0.705 | 0.540 | 2.024 | (0.702−5.835)  < | .192 |
| Weaning success (yes vs. no) | | 3.921 | 0.736 | 50.450 | (11.924−213.454) | .001 |
| **Step 5** | |  |  |  |  |  |
| Age (years) | | −0.039 | 0.026 | 0.962 | (0.914−1.012) | .134 |
| End-stage renal disease (yes vs. no) | | 1.134 | 1.328 | 3.109 | (0.230−41.966) | .393 |
| Old stroke (yes vs. no) | | −1.178 | 0.677 | 0.308 | (0.082−1.162) | .082 |
| Cause of respiratory failure | |  |  |  |  |  |
| Pulmonary | |  |  | 1 | | − |
| Cardiovascular | | −0.647 | 0.860 | 0.524 | (0.097−2.825) | .452 |
| Neurologic | | 1.494 | 1.305 | 4.456 | (0.346−57.468) | .252 |
| Post-operative | | −0.546 | 0.697 | 0.579 | (0.148−2.272) | .434 |
| Other | | −1.749 | 0.985 | 0.174 | (0.025−1.199) | .076 |
| Septic shock (yes vs. no) | | 1.493 | 0.687 | 4.448 | (1.157−17.108) | .030 |
| APACHE II at RCC transfer | | 0.149 | 0.099 | 1.161 | (0.956−1.410) | .132 |
| BMI (kg/m2) | | 0.187 | 0.068 | 1.206 | (1.055−1.378) | .006 |
| GCS | | 0.257 | 0.114 | 1.293 | (1.034−1.618) | .024 |
| Platelets (10^4^/μL) | | 0.065 | 0.025 | 1.067 | (1.015−1.121) | .011 |
| Hemoglobin (g/dL) | | 0.240 | 0.197 | 1.271 | (0.864−1.870) | .223 |
| Albumin (g/dL) | | 0.541 | 0.529 | 1.717 | (0.609−4.845) | .307 |
| Creatinine (mg/dL) | | −0.417 | 0.197 | 0.659 | (0.448−0.969) | .034 |
| DEMMI (post-rehabilitation, ≥ 20 vs. < 20) | | 1.418 | 0.805 | 4.131 | (0.852−20.018) | .078 |
| P_Emax_ (post-rehabilitation, ≥ 30 vs. < 30) (cmH_2_O) | | 0.669 | 0.536 | 1.952 | (0.683−5.583)  < | .212 |
| Weaning success (yes vs. no) | | 3.807 | 0.702 | 45.028 | (11.381−178.144) | .001 |
| **Step 6** | |  |  |  |  |  |
| Age (years) | | −0.041 | 0.026 | 0.959 | (0.912−1.009) | .107 |
| Old stroke (yes vs. no) | | −1.265 | 0.675 | 0.282 | (0.075−1.061) | .061 |
| Cause of respiratory failure | |  |  |  |  |  |
| Pulmonary | |  |  | 1 | | − |
| Cardiovascular | | −0.843 | 0.825 | 0.430 | (0.085−2.168) | .307 |
| Neurologic | | 1.342 | 1.295 | 3.827 | (0.302−48.441) | .300 |
| Post-operative | | −0.545 | 0.695 | 0.580 | (0.148−2.265) | .433 |
| Other | | −1.583 | 0.944 | 0.205 | (0.032−1.305) | .093 |
| Septic shock (yes vs. no) | | 1.386 | 0.675 | 3.998 | (1.066−15.001) | .040 |
| APACHE II at RCC transfer | | 0.151 | 0.099 | 1.163 | (0.957−1.413) | .128 |
| BMI (kg/m2) | | 0.174 | 0.065 | 1.190 | (1.048−1.353) | .008 |
| GCS | | 0.245 | 0.112 | 1.277 | (1.025−1.592) | .029 |
| Platelets (10^4^/μL) | | 0.063 | 0.025 | 1.065 | (1.013−1.118) | .013 |
| Hemoglobin (g/dL) | | 0.245 | 0.197 | 1.277 | (0.868−1.879) | .215 |
| Albumin (g/dL) | | 0.492 | 0.526 | 1.635 | (0.583−4.586) | .350 |
| Creatinine (mg/dL) | | −0.302 | 0.139 | 0.739 | (0.563−0.970) | .029 |
| DEMMI (post-rehabilitation, ≥ 20 vs. < 20) | | 1.444 | 0.802 | 4.239 | (0.880−20.417) | .072 |
| P_Emax_ (post-rehabilitation, ≥ 30 vs. < 30) (cmH_2_O) | | 0.629 | 0.529 | 1.875 | (0.665−5.290)  < | .235 |
| Weaning success (yes vs. no) | | 3.856 | 0.701 | 47.256 | (11.955−186.805) | .001 |
| **Step 7** | |  |  |  |  |  |
| Age (years) | | −0.040 | 0.025 | 0.961 | (0.915−1.009) | .111 |
| Old stroke (yes vs. no) | | −1.369 | 0.670 | 0.254 | (0.068−0.945) | .041 |
| Cause of respiratory failure | |  |  |  |  |  |
| Pulmonary | |  |  | 1 | | − |
| Cardiovascular | | −0.806 | 0.822 | 0.447 | (0.089−2.236) | .327 |
| Neurologic | | 1.473 | 1.307 | 4.361 | (0.337−54.496) | .260 |
| Post-operative | | −0.509 | 0.688 | 0.601 | (0.156−2.315) | .459 |
| Other | | −1.492 | 0.931 | 0.225 | (0.036−1.395) | .109 |
| Septic shock (yes vs. no) | | 1.263 | 0.660 | 3.534 | (0.969−12.889) | .056 |
| APACHE II at RCC transfer | | 0.135 | 0.096 | 1.145 | (0.948−1.383) | .161 |
| BMI (kg/m2) | | 0.164 | 0.062 | 1.178 | (1.044−1.330) | .008 |
| GCS | | 0.227 | 0.110 | 1.254 | (1.012−1.555) | .039 |
| Platelets (10^4^/μL) | | 0.064 | 0.025 | 1.066 | (1.016−1.119) | .010 |
| Hemoglobin (g/dL) | | 0.283 | 0.193 | 1.327 | (0.909−1.940) | .143 |
| Creatinine (mg/dL) | | −0.280 | 0.135 | 0.755 | (0.580−0.984) | .038 |
| DEMMI (post-rehabilitation, ≥ 20 vs. < 20) | | 1.456 | 0.799 | 4.289 | (0.897−20.517) | .068 |
| P_Emax_ (post-rehabilitation, ≥ 30 vs. < 30) (cmH_2_O) | | 0.685 | 0.523 | 1.983 | (0.712−5.527)  < | .190 |
| Weaning success (yes vs. no) | | 3.857 | 0.699 | 47.323 | (12.021−186.297) | .001 |
| **Step 8** | |  |  |  |  |  |
| Age (years) | | −0.037 | 0.025 | 0.964 | (0.919−1.012) | .137 |
| Old stroke (yes vs. no) | | −1.456 | 0.663 | 0.233 | (0.064−0.855) | .028 |
| Cause of respiratory failure | |  |  |  |  |  |
| Pulmonary | |  |  | 1 | | − |
| Cardiovascular | | −0.790 | 0.815 | 0.454 | (0.092−2.242) | .332 |
| Neurologic | | 1.535 | 1.358 | 4.640 | (0.324−66.388) | .258 |
| Post-operative | | −0.606 | 0.672 | 0.545 | (0.146−2.037) | .367 |
| Other | | −1.407 | 0.913 | 0.245 | (0.041−1.465) | .123 |
| Septic shock (yes vs. no) | | 1.263 | 0.648 | 3.536 | (0.993−12.588) | .051 |
| APACHE II at RCC transfer | | 0.120 | 0.093 | 1.128 | (0.939−1.354) | .198 |
| BMI (kg/m2) | | 0.176 | 0.063 | 1.192 | (1.054−1.348) | .005 |
| GCS | | 0.231 | 0.108 | 1.260 | (1.019−1.557) | .033 |
| Platelets (10^4^/μL) | | 0.066 | 0.025 | 1.069 | (1.018−1.121) | .007 |
| Hemoglobin (g/dL) | | 0.270 | 0.192 | 1.310 | (0.899−1.909) | .160 |
| Creatinine (mg/dL) | | −0.249 | 0.130 | 0.780 | (0.604−1.007) | .057 |
| DEMMI (post-rehabilitation, ≥ 20 vs. < 20) | | 1.607 | 0.783 | 4.986 | (1.074−23.150)  < | .040 |
| Weaning success (yes vs. no) | | 3.921 | 0.692 | 50.466 | (12.999−195.921) | .001 |
| **Step 9** | |  |  |  |  |  |
| Age (years) | | −0.024 | 0.022 | 0.977 | (0.935−1.020) | .282 |
| Old stroke (yes vs. no) | | −1.606 | 0.653 | 0.201 | (0.056−0.722) | .014 |
| Cause of respiratory failure | |  |  |  |  |  |
| Pulmonary | |  |  | 1 | | − |
| Cardiovascular | | −0.878 | 0.816 | 0.416 | (0.084−2.059) | .282 |
| Neurologic | | 1.679 | 1.369 | 5.360 | (0.366−78.458) | .220 |
| Post-operative | | −0.614 | 0.664 | 0.541 | (0.147−1.987) | .355 |
| Other | | −1.477 | 0.888 | 0.228 | (0.042−1.251) | .089 |
| Septic shock (yes vs. no) | | 1.459 | 0.629 | 4.300 | (1.252−14.763) | .020 |
| BMI (kg/m2) | | 0.181 | 0.064 | 1.199 | (1.058−1.359) | .005 |
| GCS | | 0.161 | 0.092 | 1.175 | (0.982−1.407) | .079 |
| Platelets (10^4^/μL) | | 0.064 | 0.024 | 1.066 | (1.017−1.118) | .008 |
| Hemoglobin (g/dL) | | 0.188 | 0.181 | 1.206 | (0.847−1.719) | .299 |
| Creatinine (mg/dL) | | −0.161 | 0.103 | 0.851 | (0.695−1.041) | .117 |
| DEMMI (post-rehabilitation, ≥ 20 vs. < 20) | | 1.421 | 0.768 | 4.140 | (0.920−18.637)  < | .064 |
| Weaning success (yes vs. no) | | 3.889 | 0.685 | 48.850 | (12.757−187.054) | .001 |
| **Step 10** | |  |  |  |  |  |
| Age (years) | | −0.021 | 0.022 | 0.979 | (0.938−1.022) | .331 |
| Old stroke (yes vs. no) | | −1.529 | 0.640 | 0.217 | (0.062−0.760) | .017 |
| Cause of respiratory failure | |  |  |  |  |  |
| Pulmonary | |  |  | 1 | | − |
| Cardiovascular | | −0.709 | 0.792 | 0.492 | (0.104−2.325) | .371 |
| Neurologic | | 1.938 | 1.364 | 6.943 | (0.480−100.496) | .155 |
| Post-operative | | −0.637 | 0.662 | 0.529 | (0.144−1.938) | .356 |
| Other | | −1.368 | 0.838 | 0.255 | (0.049−1.315) | .103 |
| Septic shock (yes vs. no) | | 1.357 | 0.620 | 3.884 | (1.153−13.082) | .029 |
| BMI (kg/m2) | | 0.180 | 0.064 | 1.197 | (1.055−1.358) | .005 |
| GCS | | 0.163 | 0.092 | 1.178 | (0.984−1.410) | .075 |
| Platelets (10^4^/μL) | | 0.066 | 0.025 | 1.068 | (1.017−1.121) | .008 |
| Creatinine (mg/dL) | | −0.172 | 0.103 | 0.842 | (0.687−1.031) | .096 |
| DEMMI (post-rehabilitation, ≥ 20 vs. < 20) | | 1.519 | 0.771 | 4.566 | (1.007−20.711)  < | .049 |
| Weaning success (yes vs. no) | | 3.851 | 0.676 | 47.033 | (12.507−176.862) | .001 |
| **Step 11** | |  |  |  |  |  |
| Old stroke (yes vs. no) | | −1.570 | 0.632 | 0.208 | (0.060−0.717) | .013 |
| Cause of respiratory failure | |  |  |  |  |  |
| Pulmonary | |  |  | 1 | | − |
| Cardiovascular | | −0.577 | 0.763 | 0.562 | (0.126−2.505) | .450 |
| Neurologic | | 2.403 | 1.370 | 11.062 | (0.755−162.065) | .079 |
| Post-operative | | −0.529 | 0.651 | 0.589 | (0.165−2.109) | .416 |
| Other | | −1.226 | 0.826 | 0.293 | (0.058−1.482) | .138 |
| Septic shock (yes vs. no) | | 1.298 | 0.601 | 3.662 | (1.128−11.886) | .031 |
| BMI (kg/m2) | | 0.179 | 0.063 | 1.196 | (1.057−1.355) | .005 |
| GCS | | 0.171 | 0.091 | 1.186 | (0.992−1.419) | .062 |
| Platelets (10^4^/μL) | | 0.067 | 0.024 | 1.070 | (1.020−1.122) | .006 |
| Creatinine (mg/dL) | | −0.152 | 0.099 | 0.859 | (0.707−1.043) | .125 |
| DEMMI (post-rehabilitation, ≥ 20 vs. < 20) | | 1.522 | 0.778 | 4.583 | (0.997−21.060)  < | .050 |
| Weaning success (yes vs. no) | | 3.760 | 0.652 | 42.956 | (11.962−154.261) | .001 |
| **Step 12** | |  |  |  |  |  |
| Old stroke (yes vs. no) | | −1.518 | 0.607 | 0.219 | (0.067−0.721) | .012 |
| Cause of respiratory failure | |  |  |  |  |  |
| Pulmonary | |  |  | 1 | | − |
| Cardiovascular | | −0.633 | 0.761 | 0.531 | (0.119−2.360) | .405 |
| Neurologic | | 2.354 | 1.364 | 10.531 | (0.727−152.537) | .084 |
| Post-operative | | −0.553 | 0.643 | 0.575 | (0.163−2.029) | .390 |
| Other | | −1.279 | 0.817 | 0.278 | (0.056−1.380) | .117 |
| Septic shock (yes vs. no) | | 1.098 | 0.569 | 2.999 | (0.984−9.140) | .053 |
| BMI (kg/m2) | | 0.159 | 0.060 | 1.173 | (1.044−1.318) | .007 |
| GCS | | 0.152 | 0.089 | 1.164 | (0.977−1.386) | .089 |
| Platelets (10^4^/μL) | | 0.064 | 0.024 | 1.066 | (1.018−1.117) | .007 |
| DEMMI (post-rehabilitation, ≥ 20 vs. < 20) | | 1.575 | 0.768 | 4.830 | (1.072−21.756)  < | .040 |
| Weaning success (yes vs. no) | | 3.658 | 0.620 | 38.788 | (11.505−130.762) | .001 |

APACHE II = Acute Physiology and Chronic Health Evaluation score; BMI = body mass index; CI = confidence interval; DEMMI = the de Morton Mobility Index; GCS = Glasgow Coma Scale; ICU = intensive care unit; P_Emax_ = maximal expiratory pressure; RCC = respiratory care center; SE = standard error.

* Variables with statistical significance (*P* < .05) in the univariate analyses (Supplementary Material Tables 1 and 2) were included in the multivariate logistic regression models. Backward variable selection was performed, and the criteria of *P* values for entry and stay were set at .05 and .10, respectively.
