# Supplementary material for: Functional Status After Pulmonary Rehabilitation as a Predictor of Weaning Success and Survival in Patients Requiring Prolonged Mechanical Ventilation
Source: Front Med (Lausanne). 2021 Jun 2;8:675103. doi: 10.3389/fmed.2021.675103 (PMC8206270; doi:10.3389/fmed.2021.675103)
Supplement: Supplementary file 8 [file Table_8.DOC]

**Supplementary Material Table 8**. Details of backward variable selection in the multivariate Cox regression models for significant clinical characteristics associated with overall survival after respiratory failure*

| **Parameters** | **Hazard ratio (95% CI)** | | ***P*** |
| --- | --- | --- | --- |
| **Step 1** |  |  |  |
| Age (years) | 1.024 | (0.998−1.050) | .066 |
| Chronic kidney disease (yes vs. no) | 0.863 | (0.389−1.910) | .715 |
| End-stage renal disease (yes vs. no) | 2.154 | (0.702−6.606) | .180 |
| Old stroke (yes vs. no) | 1.966 | (0.969−3.988) | .061 |
| Other neurologic disease (yes vs. no) | 1.072 | (0.494−2.327) | .861 |
| Cancer (yes vs. no) | 1.255 | (0.628−2.510) | .520 |
| APACHE II at ICU admission | 0.996 | (0.956−1.037) | .832 |
| Septic shock (yes vs. no) | 0.775 | (0.404−1.487) | .443 |
| APACHE II at RCC transfer | 0.940 | (0.848−1.042) | .240 |
| GCS | 0.901 | (0.802−1.012) | .080 |
| Platelets (10^4^/μL) | 0.969 | (0.944−0.995) | .019 |
| Hemoglobin (g/dL) | 0.970 | (0.782−1.202) | .780 |
| Albumin (g/dL) | 0.870 | (0.474−1.591) | .650 |
| Bilirubin (mg/dL) | 1.062 | (1.004−1.123) | .037 |
| Creatinine (mg/dL) | 1.114 | (0.912−1.360) | .292 |
| Phosphate (mg/dL) | 1.078 | (0.884−1.315) | .458 |
| DEMMI (post-rehabilitation, ≥ 20 vs. < 20) | 0.215 | (0.063−0.733) | .014 |
| P_Emax_ (post-rehabilitation, ≥ 30 vs. < 30) (cmH_2_O) | 0.947 | (0.524−1.714) | .858  < |
| Weaning success (yes vs. no) | 0.164 | (0.089−0.302) | .001 |
| **Step 2** |  |  |  |
| Age (years) | 1.024 | (0.998−1.050) | .065 |
| Chronic kidney disease (yes vs. no) | 0.860 | (0.389−1.900) | .708 |
| End-stage renal disease (yes vs. no) | 2.147 | (0.701−6.579) | .181 |
| Old stroke (yes vs. no) | 1.951 | (0.967−3.935) | .062 |
| Cancer (yes vs. no) | 1.268 | (0.640−2.511) | .496 |
| APACHE II at ICU admission | 0.996 | (0.958−1.036) | .853 |
| Septic shock (yes vs. no) | 0.781 | (0.409−1.490) | .453 |
| APACHE II at RCC transfer | 0.940 | (0.848−1.042) | .239 |
| GCS | 0.898 | (0.804−1.003) | .057 |
| Platelets (10^4^/μL) | 0.970 | (0.945−0.995) | .018 |
| Hemoglobin (g/dL) | 0.969 | (0.782−1.202) | .778 |
| Albumin (g/dL) | 0.872 | (0.478−1.593) | .657 |
| Bilirubin (mg/dL) | 1.061 | (1.003−1.122) | .037 |
| Creatinine (mg/dL) | 1.115 | (0.913−1.361) | .284 |
| Phosphate (mg/dL) | 1.075 | (0.884−1.306) | .470 |
| DEMMI (post-rehabilitation, ≥ 20 vs. < 20) | 0.215 | (0.063−0.733) | .014 |
| P_Emax_ (post-rehabilitation, ≥ 30 vs. < 30) (cmH_2_O) | 0.937 | (0.525−1.674) | .827  < |
| Weaning success (yes vs. no) | 0.164 | (0.089−0.301) | .001 |
| **Step 3** |  |  |  |
| Age (years) | 1.023 | (0.999−1.049) | .065 |
| Chronic kidney disease (yes vs. no) | 0.862 | (0.390−1.905) | .713 |
| End-stage renal disease (yes vs. no) | 2.158 | (0.703−6.619) | .179 |
| Old stroke (yes vs. no) | 1.935 | (0.964−3.884) | .063 |
| Cancer (yes vs. no) | 1.263 | (0.639−2.500) | .502 |
| Septic shock (yes vs. no) | 0.785 | (0.413−1.495) | .462 |
| APACHE II at RCC transfer | 0.940 | (0.848−1.042) | .242 |
| GCS | 0.899 | (0.806−1.003) | .057 |
| Platelets (10^4^/μL) | 0.970 | (0.946−0.995) | .017 |
| Hemoglobin (g/dL) | 0.974 | (0.790−1.201) | .805 |
| Albumin (g/dL) | 0.877 | (0.481−1.597) | .667 |
| Bilirubin (mg/dL) | 1.061 | (1.003−1.122) | .038 |
| Creatinine (mg/dL) | 1.113 | (0.913−1.356) | .290 |
| Phosphate (mg/dL) | 1.074 | (0.884−1.304) | .474 |
| DEMMI (post-rehabilitation, ≥ 20 vs. < 20) | 0.214 | (0.063−0.727) | .014 |
| P_Emax_ (post-rehabilitation, ≥ 30 vs. < 30) (cmH_2_O) | 0.943 | (0.529−1.679) | .841  < |
| Weaning success (yes vs. no) | 0.166 | (0.091−0.300) | .001 |
| **Step 4** |  |  |  |
| Age (years) | 1.023 | (0.999−1.049) | .065 |
| Chronic kidney disease (yes vs. no) | 0.847 | (0.390−1.838) | .675 |
| End-stage renal disease (yes vs. no) | 2.180 | (0.715−6.644) | .170 |
| Old stroke (yes vs. no) | 1.958 | (0.984−3.896) | .056 |
| Cancer (yes vs. no) | 1.260 | (0.637−2.489) | .506 |
| Septic shock (yes vs. no) | 0.782 | (0.412−1.486) | .453 |
| APACHE II at RCC transfer | 0.942 | (0.851−1.043) | .247 |
| GCS | 0.899 | (0.806−1.003) | .057 |
| Platelets (10^4^/μL) | 0.970 | (0.946−0.994) | .015 |
| Hemoglobin (g/dL) | 0.973 | (0.790−1.199) | .800 |
| Albumin (g/dL) | 0.871 | (0.478−1.587) | .653 |
| Bilirubin (mg/dL) | 1.062 | (1.005−1.122) | .034 |
| Creatinine (mg/dL) | 1.111 | (0.912−1.354) | .295 |
| Phosphate (mg/dL) | 1.073 | (0.884−1.304) | .475 |
| DEMMI (post-rehabilitation, ≥ 20 vs. < 20) | 0.211 | (0.062−0.715) | .012  < |
| Weaning success (yes vs. no) | 0.165 | (0.091−0.297) | .001 |
| **Step 5** |  |  |  |
| Age (years) | 1.023 | (0.998−1.048) | .067 |
| Chronic kidney disease (yes vs. no) | 0.854 | (0.394−1.850) | .689 |
| End-stage renal disease (yes vs. no) | 2.159 | (0.711−6.559) | .174 |
| Old stroke (yes vs. no) | 1.942 | (0.980−3.848) | .057 |
| Cancer (yes vs. no) | 1.288 | (0.668−2.487) | .450 |
| Septic shock (yes vs. no) | 0.804 | (0.440−1.470) | .479 |
| APACHE II at RCC transfer | 0.943 | (0.852−1.043) | .256 |
| GCS | 0.899 | (0.806−1.002) | .055 |
| Platelets (10^4^/μL) | 0.970 | (0.946−0.994) | .015 |
| Albumin (g/dL) | 0.863 | (0.476−1.565) | .628 |
| Bilirubin (mg/dL) | 1.062 | (1.005−1.123) | .032 |
| Creatinine (mg/dL) | 1.113 | (0.915−1.355) | .283 |
| Phosphate (mg/dL) | 1.073 | (0.884−1.302) | .479 |
| DEMMI (post-rehabilitation, ≥ 20 vs. < 20) | 0.209 | (0.062−0.705) | .012  < |
| Weaning success (yes vs. no) | 0.165 | (0.091−0.298) | .001 |
| **Step 6** |  |  |  |
| Age (years) | 1.021 | (0.998−1.045) | .072 |
| End-stage renal disease (yes vs. no) | 2.072 | (0.699−6.137) | .189 |
| Old stroke (yes vs. no) | 1.894 | (0.964−3.722) | .064 |
| Cancer (yes vs. no) | 1.305 | (0.678−2.511) | .426 |
| Septic shock (yes vs. no) | 0.827 | (0.460−1.487) | .526 |
| APACHE II at RCC transfer | 0.941 | (0.851−1.042) | .243 |
| GCS | 0.898 | (0.805−1.002) | .054 |
| Platelets (10^4^/μL) | 0.971 | (0.949−0.994) | .015 |
| Albumin (g/dL) | 0.845 | (0.470−1.518) | .574 |
| Bilirubin (mg/dL) | 1.061 | (1.005−1.121) | .033 |
| Creatinine (mg/dL) | 1.094 | (0.916−1.305) | .321 |
| Phosphate (mg/dL) | 1.071 | (0.883−1.299) | .487 |
| DEMMI (post-rehabilitation, ≥ 20 vs. < 20) | 0.204 | (0.061−0.686) | .010  < |
| Weaning success (yes vs. no) | 0.166 | (0.092−0.299) | .001 |
| **Step 7** |  |  |  |
| Age (years) | 1.022 | (0.999−1.046) | .064 |
| End-stage renal disease (yes vs. no) | 2.036 | (0.695−5.963) | .195 |
| Old stroke (yes vs. no) | 1.934 | (0.985−3.796) | .055 |
| Cancer (yes vs. no) | 1.289 | (0.669−2.481) | .448 |
| Septic shock (yes vs. no) | 0.854 | (0.479−1.521) | .591 |
| APACHE II at RCC transfer | 0.950 | (0.863−1.045) | .290 |
| GCS | 0.903 | (0.810−1.005) | .062 |
| Platelets (10^4^/μL) | 0.970 | (0.948−0.992) | .009 |
| Bilirubin (mg/dL) | 1.062 | (1.005−1.121) | .032 |
| Creatinine (mg/dL) | 1.091 | (0.915−1.301) | .331 |
| Phosphate (mg/dL) | 1.067 | (0.879−1.296) | .513 |
| DEMMI (post-rehabilitation, ≥ 20 vs. < 20) | 0.205 | (0.061−0.687) | .010  < |
| Weaning success (yes vs. no) | 0.165 | (0.092−0.297) | .001 |
| **Step 8** |  |  |  |
| Age (years) | 1.022 | (0.999−1.046) | .058 |
| End-stage renal disease (yes vs. no) | 2.145 | (0.744−6.187) | .158 |
| Old stroke (yes vs. no) | 1.864 | (0.965−3.601) | .064 |
| Cancer (yes vs. no) | 1.322 | (0.694−2.518) | .396 |
| APACHE II at RCC transfer | 0.942 | (0.860−1.032) | .202 |
| GCS | 0.898 | (0.808−0.998) | .045 |
| Platelets (10^4^/μL) | 0.971 | (0.949−0.993) | .010 |
| Bilirubin (mg/dL) | 1.062 | (1.006−1.122) | .029 |
| Creatinine (mg/dL) | 1.087 | (0.912−1.295) | .352 |
| Phosphate (mg/dL) | 1.079 | (0.892−1.305) | .432 |
| DEMMI (post-rehabilitation, ≥ 20 vs. < 20) | 0.204 | (0.061−0.683) | .010  < |
| Weaning success (yes vs. no) | 0.169 | (0.094−0.303) | .001 |
| **Step 9** |  |  |  |
| Age (years) | 1.021 | (0.998−1.044) | .072 |
| End-stage renal disease (yes vs. no) | 1.979 | (0.697−5.618) | .200 |
| Old stroke (yes vs. no) | 1.975 | (1.044−3.738) | .037 |
| Cancer (yes vs. no) | 1.309 | (0.685−2.501) | .416 |
| APACHE II at RCC transfer | 0.938 | (0.856−1.027) | .167 |
| GCS | 0.888 | (0.802−0.983) | .022 |
| Platelets (10^4^/μL) | 0.970 | (0.948−0.992) | .008 |
| Bilirubin (mg/dL) | 1.059 | (1.005−1.117) | .033 |
| Creatinine (mg/dL) | 1.122 | (0.962−1.309) | .141 |
| DEMMI (post-rehabilitation, ≥ 20 vs. < 20) | 0.211 | (0.063−0.704) | .011  <  < |
| Weaning success (yes vs. no) | 0.167 | (0.093−0.299) | .001 |
| **Step 10** |  |  |  |
| Age (years) | 1.022 | (0.9998−1.045) | .052 |
| End-stage renal disease (yes vs. no) | 2.119 | (0.754−5.954) | .154 |
| Old stroke (yes vs. no) | 2.047 | (1.087−3.854) | .027 |
| APACHE II at RCC transfer | 0.941 | (0.860−1.030) | .190 |
| GCS | 0.893 | (0.807−0.988) | .028 |
| Platelets (10^4^/μL) | 0.970 | (0.948−0.992) | .008 |
| Bilirubin (mg/dL) | 1.058 | (1.004−1.115) | .034 |
| Creatinine (mg/dL) | 1.105 | (0.951−1.284) | .194 |
| DEMMI (post-rehabilitation, ≥ 20 vs. < 20) | 0.211 | (0.063−0.705) | .012 |
| Weaning success (yes vs. no) | 0.162 | (0.091−0.290) | .001 |
| **Step 11** |  |  |  |
| Age (years) | 1.023 | (0.9996−1.046) | .054 |
| End-stage renal disease (yes vs. no) | 2.835 | (1.135−7.081) | .026 |
| Old stroke (yes vs. no) | 1.983 | (1.052−3.739) | .034 |
| APACHE II at RCC transfer | 0.960 | (0.881−1.045) | .345 |
| GCS | 0.907 | (0.823−0.9997) | .049 |
| Platelets (10^4^/μL) | 0.972 | (0.950−0.994) | .015 |
| Bilirubin (mg/dL) | 1.061 | (1.007−1.119) | .027 |
| DEMMI (post-rehabilitation, ≥ 20 vs. < 20) | 0.221 | (0.066−0.739) | .014  < |
| Weaning success (yes vs. no) | 0.159 | (0.089−0.282) | .001 |
| **Step 12** |  |  |  |
| Age (years) | 1.021 | (0.998−1.045) | .080 |
| End-stage renal disease (yes vs. no) | 2.368 | (1.034−5.420) | .041 |
| Old stroke (yes vs. no) | 1.974 | (1.047−3.723) | .036 |
| GCS | 0.929 | (0.856−1.008) | .076 |
| Platelets (10^4^/μL) | 0.974 | (0.953−0.995) | .017 |
| Bilirubin (mg/dL) | 1.062 | (1.008−1.119) | .025 |
| DEMMI (post-rehabilitation, ≥ 20 vs. < 20) | 0.237 | (0.072−0.785) | .018  < |
| Weaning success (yes vs. no) | 0.168 | (0.096−0.293) | .001 |

APACHE II = Acute Physiology and Chronic Health Evaluation score; CI = confidence interval; DEMMI = the de Morton Mobility Index; GCS = Glasgow Coma Scale; ICU = intensive care unit; P_Emax_ = maximal expiratory pressure; RCC = respiratory care center.

*Variables with statistical significance (*P* < .05) in the univariate analyses (Supplementary Material Tables 3) were included in the multivariate Cox regression models. Backward variable selection was performed, and the criteria of *P* values for entry and stay were set at .05 and .10, respectively.
