# Supplementary figures and images for: Functional Status After Pulmonary Rehabilitation as a Predictor of Weaning Success and Survival in Patients Requiring Prolonged Mechanical Ventilation
Source: Front Med (Lausanne). 2021 Jun 2;8:675103. doi: 10.3389/fmed.2021.675103 (PMC8206270; doi:10.3389/fmed.2021.675103)

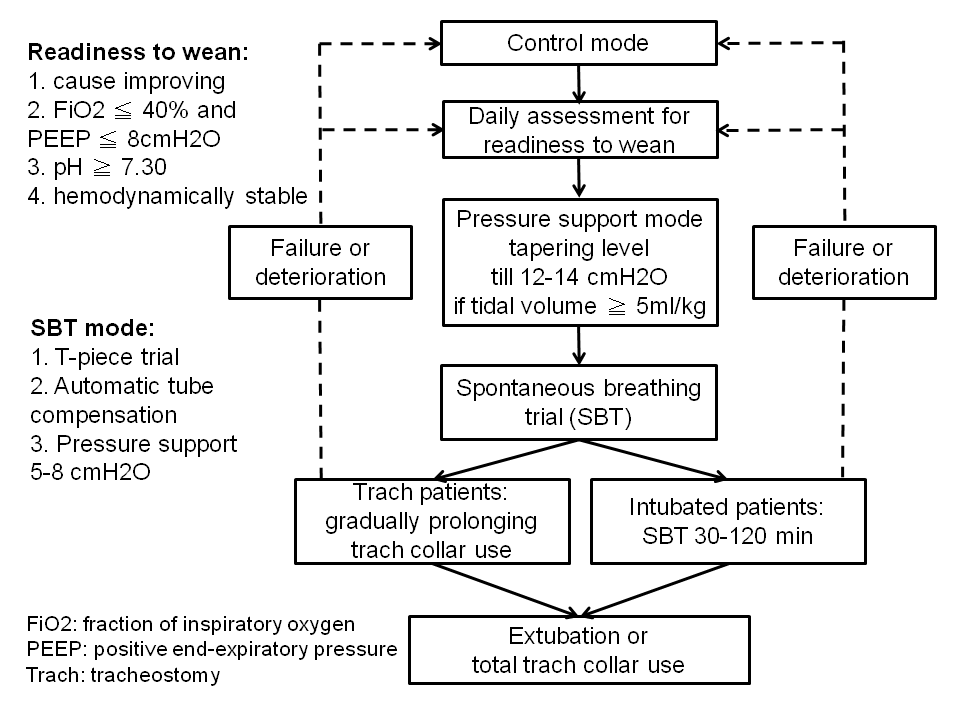

Supplement: Supplementary file 9 [file Image_1.TIF]
